# Continuity of care for people with hypertension in primary health care: a scoping review

**DOI:** 10.1590/1980-220X-REEUSP-2025-0053en

**Published:** 2026-01-09

**Authors:** Amanda Maria Villas Bôas Ribeiro, Patty Fidelis de Almeida, Ana Luiza Queiroz Vilasbôas

**Affiliations:** 1 Universidade Federal da Bahia, Instituto de Saúde Coletiva, Salvador, BA, Brazil.; 2 Universidade Federal Fluminense, Instituto de Saúde Coletiva, Niteroi, RJ, Brazil.

**Keywords:** Hypertension, Continuity of Patient Care, Primary Health Care

## Abstract

**Objective:**

To map the evidence on continuity of care for people with systemic arterial hypertension in Primary Health Care (PHC), pointing out limitations and strategies for its implementation.

**Method:**

Scope review, without time limits, based on the Web of Science, PubMed, Latin American and Caribbean Health Sciences Literature, and Scientific Electronic Library Online databases. Theses and dissertations from the Brazilian Digital Library and the Catalog of the Coordination for the Improvement of Higher Education Personnel were included. The selection of studies was performed by two independent blind reviewers, and any disagreements were resolved by a third reviewer, resulting in a corpus of 32 studies. Data extraction was performed based on an adaptation of the JBI instrument, covering information on the studies, methodology, and recommendations for continuity of care. The data were organized in an Excel spreadsheet and analyzed thematically, being interpreted in light of the dimensions of continuity of care.

**Results:**

Continuity of care in hypertension in PHC improves blood pressure control, reducing morbidity, mortality, and health costs. Patients followed by the same health professional show better adherence to treatment and quality of life. Limitations were identified, such as staff turnover, poor communication between services, and the absence of consolidated care flows. Strategies include strengthening teamwork, interprofessional coordination, the use of shared electronic medical records, and the adoption of formal referral and counter-referral flows.

**Conclusions:**

Implementing team-based care and promoting the sharing of activities among professionals are strategies for achieving continuity of care.

## INTRODUCTION

Over the last three decades, the number of people with systemic arterial hypertension (SAH) worldwide has risen from 650 million in 1990 to 1.3 billion in 2019, with estimates of global prevalence of 49% among men and women aged 50 to 79^(1)^, highlighting its magnitude. Given this problem, it is essential to ensure adherence to drug and non-drug therapy through access to health services, good relationships with professionals^(2)^, and continuity of care to prevent risks and complications arising from SAH, especially the occurrence of myocardial ischemia, chronic kidney disease, and stroke.

Primary Health Care (PHC), which is territorially based and community-oriented, plays a crucial role in the control of chronic noncommunicable diseases, such as SAH, and should ensure timely and adequate access to the Health Care Network (HCN) services, coordinating care and linking community and assistance resources to ensure continuity of health care^(3)^.

However, individuals with SAH face difficulties in accessing health services^(4,5)^, including PHC, despite its importance for disease control and reduction of deaths^(6,7,8)^. The challenges experienced range from restricted and fragmented access to care^(9)^, lack of material resources and availability of medications^(6)^, to the absence of communication between health service professionals^(10)^.

In view of these evident obstacles, there is a clear social importance in expanding the discussion on continuity of care to create appropriate strategies for its implementation in the HCN under the coordination of PHC, since regular and continuous monitoring of patients with SAH reduces the chances of serious complications and improves therapeutic adherence, self-care, and quality of life^(11)^, reduces the burden on medium and high complexity care with hospitalizations, tests, and consultations^(12,13,14,15)^.

Continuity of care is often confused with longitudinality, coordination, or even comprehensive care^(16)^. However, although these concepts are related, they have differences. Continuity is considered to be the provision of integrated, coordinated, and sequential care over time, based on the establishment of a bond and trust, centered on the patient’s health needs, with the management of clinical information shared by different health professionals and services, under the coordination of PHC^(16,17)^. It is worth noting that continuity is linked to the individual experience of each user in the course of their therapeutic project related to a specific health problem^(18)^, such as SAH.

The objective of this article is to map the evidence on the development of continuity of care for people diagnosed with SAH by PHC, pointing out limitations and strategies for its implementation. A preliminary search was conducted using the Medical Subject Headings (MeSH) “care continuity” and “hypertension” in the Open Science Framework, Joanna Briggs Institute Evidence Synthesis, and no studies or scope review protocols on the subject were found, which justifies the relevance of this article.

## METHOD

### Study Design

This is a scoping review conducted according to the method recommended by the Joanna Briggs Institute (JBI)^(19)^ and presented according to the recommendations of the Preferred Reporting Items for Systematic reviews and Meta-Analyses extension for Scoping Reviews (PRISMA-ScR) checklist^(20)^.

This type of research consists of an exploratory review aimed at mapping the relevant literature in the area of interest and its key concepts, as well as identifying gaps in the scientific literature that help define research questions^(21)^. The steps of the scoping review include: formulating the research question and objective; identifying relevant documents that are in line with the scope and breadth of the review’s purposes; selecting studies according to predefined criteria; mapping data; summarizing results through qualitative thematic analysis in relation to the objective and question; and presenting the results^(19)^.

The protocol for this review, outlined based on the research question, was registered on the Open Science Framework platform for registering scientific works via the link osf.io/tdr6y.

### Guiding Question

The research question was outlined based on the Problem, Concept, and Context (PCC) mnemonic strategy. This methodology helps to identify key topics to guide data collection.

Participants (P) were defined as people diagnosed with SAH. With regard to Concept (C), studies addressing the continuity of care for people with Systemic Arterial Hypertension (SAH) were included in this review. The concept of continuity of care involves the relationship established between the healthcare professional and the patient that extends beyond episodes of illness, being considered a “reflection of a sense of affiliation, often expressed in terms of an implicit contract of loyalty on the part of the patient and clinical responsibility on the part of the physician,” with access to health services and coordination of care as a facilitating agent as a prerequisite^(17)^. In turn, with regard to Context (C), studies conducted in Primary Health Care were selected, with national, regional, municipal, district, or local coverage.

Thus, reconciling the key topics of the PCC and the objective of the study, the research question is: How is continuity of care for people diagnosed with SAH developed by PHC?

### Search Strategies

This review was conducted in the following databases: Web of Science Core Collection, PubMed via the National Library of Medicine/The National Center for Biotechnology Information (NLM/NCBI), Latin American and Caribbean Health Sciences Literature (LILACS) via the Virtual Health Library, and the Scientific Electronic Library Online (SciELO). The search for gray literature was performed through the Brazilian Digital Library of Theses and Dissertations and the CAPES Catalog.

The study used free terms associated with the Medical Subject Headings (MeSH) descriptors and Health Sciences Descriptors (DeCS): Primary Health Care, Hypertension, and Continuity of patient care. The search strategies followed the definitions of the corresponding database and were developed with the support of a librarian specialized in reviews. Data collection was performed in January 2024.

### Eligibility Criteria

As recommended^(22)^, inclusion and exclusion criteria were defined for the selection of articles. Thus, the sources of evidence for this review were full texts of primary studies, theses, and dissertations, all published in health databases. Studies addressing continuity of care for people with SAH were included, with documents available in full, published in Portuguese, Spanish, or English, with no defined time limits, in order to retrieve as many publications related to the research question as possible. Review articles, review study protocols, opinion articles, conference proceedings, comments, editorials, and book chapters were not included.

### Data Extraction

The articles found in the search were grouped and imported into the Rayyan Intelligent Systematic Review tool to exclude duplicates and select the studies. Next, two reviewers independently and blindly selected the sources of evidence based on reading the titles and abstracts. Disagreements were resolved by a third reviewer, and the reasons for excluding manuscripts were justified. Subsequently, studies that met the criteria were evaluated in full for a complete assessment of the retained material and composition of the final sample for data extraction.

Data extraction was organized based on the adaptation of the instrument developed by JBI^(20)^ to meet the objectives of this study, containing information regarding the identification of authors, full title, year of publication, objectives, methodology (type of study, methods adopted, participants and/or data sources, setting, scope), summary of results, difficulties and facilities for developing continuity of care for people with SAH in the PHC setting, and study recommendations for achieving continuity of care. All stages of data extraction from the selected studies were performed using an Excel spreadsheet.

### Data Analysis and Presentation

To describe the results of the search and selection of studies, we used the identification, screening, and inclusion process flowchart adapted from the Preferred Reporting Items for Systematic Review and Meta-analyses (PRISMA-ScR)^(19)^.

After selecting the studies, the extracted data were organized in an adapted collection instrument^(20)^, containing information such as authors, year, country, objectives, methodological design, main findings, and aspects related to continuity of care. Data analysis was conducted through thematic content analysis^(23)^, applied systematically to qualitative and quantitative data.

In quantitative studies, numerical findings were interpreted in light of their meanings and implications described in the articles themselves, being categorized according to emerging thematic dimensions, such as barriers, effects, and strategies associated with continuity of care. Thus, quantitative data were not analyzed statistically but integrated into the qualitative analysis based on their interpretive content, according to their relevance to the object of this study.

The analysis process followed the following steps: (1) skimming of the full texts; (2) coding of the data extracted in the collection instrument; (3) categorization of the contents by thematic similarity; (4) identification of central themes related to the object of the review, especially those dealing with the limits, effects, and strategies for effective continuity of care.

The results of the analysis were organized and presented in explanatory tables, with narrative summaries, and interpreted in light of the dimensions of continuity of care^(17)^. Continuity of care can be understood from three main dimensions: managerial, informational, and relational. Managerial continuity refers to the management of care over time and across different points in the healthcare network, requiring coordination between services and clinical management appropriate to the user’s needs^(17,18)^. Informational continuity enables the effective sharing of relevant information about the user’s history, context, and healthcare between professionals and services^(16,17)^. Finally, relational continuity involves establishing lasting therapeutic bonds between users and health professionals, sustaining trust, welcoming, and the perception of continuous care over time^(17,18)^.

### Ethical Aspects

The data used are publicly available, so there was no need to submit them to the Research Ethics Committee.

## RESULTS

Initially, 230 studies were identified, including 214 articles in databases and 16 dissertations and theses. Of these studies, 75 were duplicates and were therefore removed. After reading the titles and abstracts by two researchers in a double-blind manner, 89 studies were excluded because they did not address the theme or did not meet the established criteria. A total of 66 studies were read in full, of which 34 were excluded: two because they were not available in full, seven because of the type of publication (proceedings, study protocols, and therapeutic guidelines), 22 because they did not answer the research question, two because they did not address the population in question, and one because it was unrelated to the concept of continuity of care.

**Figure 1:**
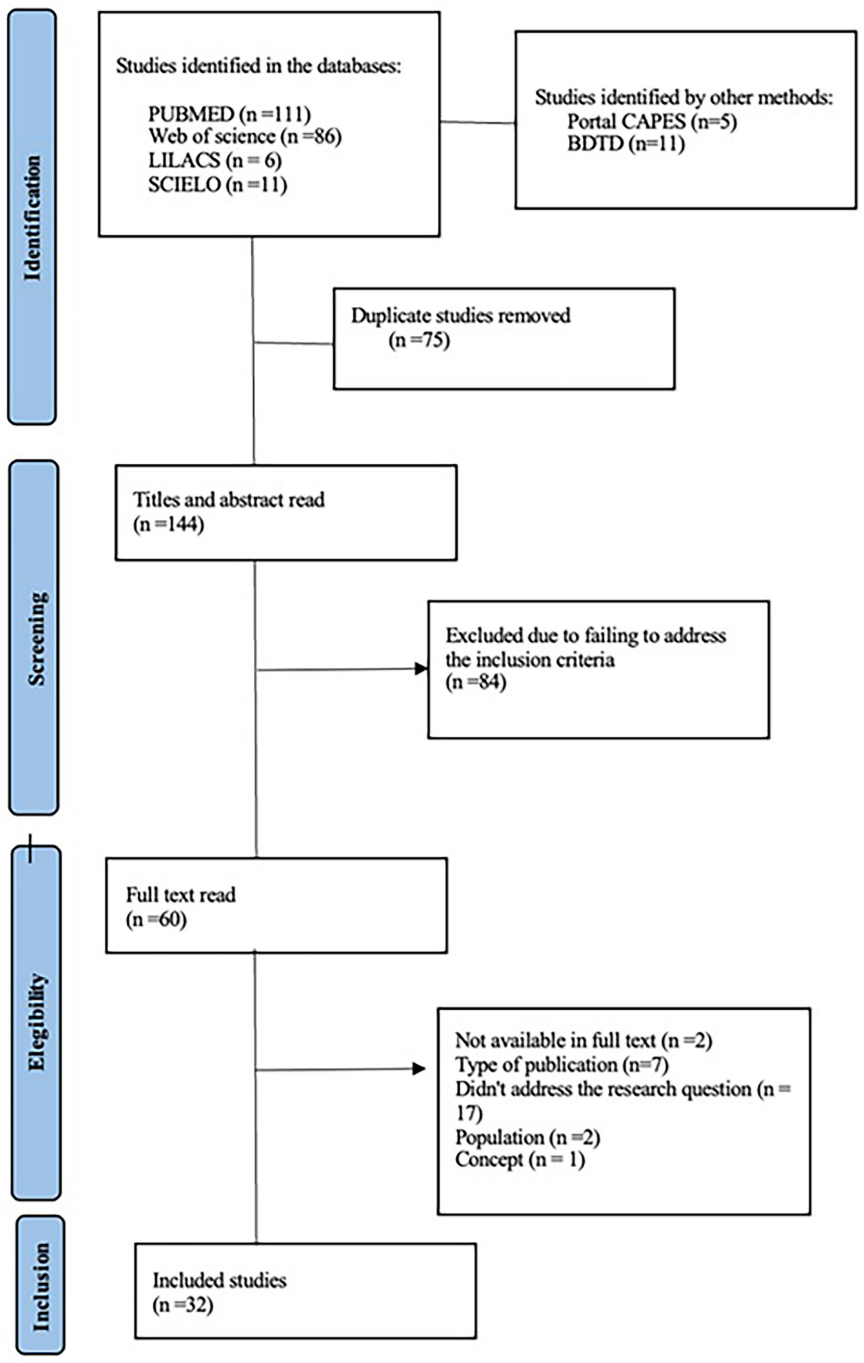
Flowchart of the selection process for studies on continuity of care for people with SAH in the context of Primary Health Care.

Thus, the final sample consisted of 32 studies, including 20 scientific articles and 12 theses and dissertations. In order to systematize the study selection process, PRISMA-SrC^(20)^ was used (Figure 1).

Next, we sought to present the characterization of the studies analyzed and a summary of the findings based on two broad categories identified from the content analysis: effects of continuity in healthcare for people with SAH and challenges in developing continuity of care for patients with SAH in PHC.

### Characterization of the Studies

The temporal dimension of the studies corresponded to the period between 2005 and 2023, as shown in Chart 1. There was a gradual increase in publications from 2019 onwards, with approximately 52.94% of studies published between 2019 and 2023. Quantitative studies were predominant (18 studies), especially cross-sectional ones (10 cases).

**Chart 1:** Characterization of selected studies according to objectives, methodology, language, and year of publication – Niterói, RJ, Brasil, 2025.

Authors	Country	Objective	Methodology
Inkster et al.^(24)^	Scotland	Investigate aspects of hypertension management in relation to sociodemographic variables, antihypertensive drug treatment, and organizational factors in primary care.	Quantitative, observational study conducted with 560 randomly selected patients aged between 40 and 79 years undergoing treatment for hypertension. Data relating to the care process were collected.
Cunha^(25)^	Brazil	Investigate compliance with the longitudinal link attribute when experimenting with different organizational models of primary health care in the context of the Brazilian Unified Health System (SUS).	Qualitative study conducted through semi-structured interviews with professionals, review of a sample of medical records, and application of a survey to a sample of users.
Malfatti and Assunção^(8)^	Brazil	Assess the annual average of diabetes and hypertension registrations and the corresponding level of patient follow-up by municipalities.	Cross-sectional study using diabetes and hypertension records and follow-up by health professionals from primary health care teams in municipalities in a health region of Rio Grande do Sul.
Machado^(26)^	Brazil	Assess the degree of integration of the health services network in caring for people with SAH.	Evaluative study conducted with managers and technical staff from primary care, surveillance, cardiology, urgent and emergency care, psychosocial care, hospital network, and regulation.
Martins^(27)^	Brazil	Analyze access to and continuity of care for patients with hypertension and diabetes mellitus.	Mixed study, with document analysis, telephone survey, and semi-structured interviews. A comparative analysis was performed according to the type of health unit: Family Doctor Program Units, Basic Units, and Polyclinics.
Sousa et al.^(9)^	Brazil	Analyze the conditions for full access to the healthcare network, considering care at a Family Health Unit through to specialized care.	Mixed methods study, using focus groups with PHC professionals and data collection from the Health Regulation Center System and the Appointment Scheduling System.
Katz et al.^(10)^	Canada	Analyze modes of access to healthcare for adults with chronic diseases.	Cohort study conducted with 347,606 patients who had at least four outpatient visits with a primary care physician or specialist during the study period.
Hanafi et al.^(28)^	Malasia	Investigate the relationship between personal continuity and blood pressure control among patients with hypertension at an academic primary care center.	Retrospective longitudinal cohort study conducted by reviewing 1,060 medical records of adult patients with hypertension.
Li et al.^(29)^	China	Compare perceived quality of primary care in terms of accessibility, continuity, coordination, and comprehensiveness among hypertensive patients.	Cross-sectional study using the adapted and validated Chinese version of the Primary Care Assessment Tool (PCAT).
Li et al.^(30)^	China	Compare continuity of care among hypertensive patients between the Direct Management Model of community health centers and the Loose Collaboration Model.	Cross-sectional study conducted in four randomly selected health centers in China, with 386 patients in Nanjing and 396 in Wuhan.
Baricati^(31)^	Brazil	Understanding the longitudinal nature of care based on the experiences of PHC users.	Qualitative, descriptive, and exploratory study conducted in the urban area of a municipality.
Li et al.^(32)^	China	Investigate changes in the perceived quality of primary care by elderly hypertensive patients.	Cross-sectional study conducted in two phases with patients treated at Primary Health Care centers in Shanghai.
Wollum et al.^(33)^	South Africa	Identify basic characteristics and barriers to the treatment of hypertension, hypercholesterolemia, and diabetes.	Mixed methods study with surveys in PHC units, focus group discussions, and interviews with key informants.
Santos^(34)^	Brazil	Evaluate the coordination between primary care and specialized care services in the care of hypertensive patients.	Quantitative-qualitative study conducted with primary health care physicians.
Khanam et al.^(35)^	Australia	Check the relationship between blood pressure control and continuity of care.	Quantitative study, data collected from 329 general practices located across Australia.
Lall et al.^(36)^	India	Understanding how primary care for diabetes and hypertension is organized in Indian health facilities.	Mixed methods study with in-depth interviews with 24 patients with diabetes and hypertension and six physicians in three public health units and three private units.
Silva^(37)^	Brazil	To evaluate the effect of case management on blood pressure and complications in adults with hypertension.	Randomized clinical trial conducted at a primary care health facility.
Chukwuma et al.^(38)^	Tajikistan	Understand gaps in continuity of care in PHC, challenges, and propose solutions.	Mixed methods study. Focus groups with service users, health care providers, and administrators.
Leniz and Gulliford^(39)^	Chile	Explore factors related to continuity of care and their association with diabetes and hypertension care and disease control.	Cross-sectional study based on data from the National Health Survey conducted in Chile with PHC users.
Neves^(40)^	Brazil	Analyze how relationships are formed among Family Health Strategy professionals regarding care for users with hypertension and diabetes.	Descriptive study with a qualitative and quantitative approach.
Barrera et al.^(41)^	Colombia	Identify the conditions associated with achieving controlled high blood pressure.	Longitudinal observational study with individuals diagnosed with hypertension treated at two primary care centers.
Fraser-Hurt et al.^(6)^	Samoa	Assess problems in continuity of care and identify possible solutions, using hypertension as a marker condition.	Mixed methods study, using data from household surveys of adults integrated with data from medical records of patients with hypertension and results from focus group discussions with patients and health professionals.
Xu et al.^(42)^	Hong Kong	Investigate the effectiveness of continuity of team-based care in preventing cardiovascular disease and mortality in patients with hypertension.	Retrospective cohort study conducted with patients who visited primary care in Hong Kong from 2008 to 2018.
Bellas et al.^(43)^	Brazil	Evaluate resilient performance in terms of continuity of care and longitudinal care for systemic arterial hypertension and diabetes mellitus by primary care units during the COVID-19 pandemic.	Mixed methods study, using quantitative and qualitative approaches. It used secondary data from the Information System and in-depth interviews with managers and technical professionals from the program area coordination department of the municipality of Rio de Janeiro, Brazil.
Almalki et al.^(44)^	Saudi Arabia	Explore continuity of care and its predictors in primary health care settings among patients with chronic diseases in Saudi Arabia.	Cross-sectional study conducted with 193 PHC users with chronic diseases who had at least four previous visits to primary care units in the last year.
Trivedi et al.^(45)^	Australia	Explore factors that may influence blood pressure control in patients with atrial fibrillation and hypertension.	Retrospective, cross-sectional study. A database containing electronic health records from general clinics was used.
Dehghani Tafti et al.^(46)^	Iran	Determine the continuity of care for patients with chronic conditions such as hypertension and diabetes during the COVID-19 pandemic.	Retrospective cross-sectional study. The study population consisted of all patients with type 2 diabetes and hypertension who had an active medical record at one of the health centers for at least two years prior to the COVID-19 pandemic.
Damião^(47)^	Brazil	Analyze the impact of the pandemic on the continuity of care for patients with hypertension and diabetes mellitus in the municipality of Pinhais, Paraná.	Longitudinal, retrospective study involving 540 patients with hypertension or diabetes, monitored by Family Health Units.
Cardoso^(48)^	Brazil	Analyze the prioritization of care in the fight against COVID-19 in relation to systematic care for hypertensive patients, diabetics, pregnant women, and children in Primary Care in the Federal District.	Cross-sectional study analyzing data from hypertensive, diabetic, pregnant, and pediatric patients monitored in primary care from 2018 to 2020 in the Federal District.
Cavalcante^(49)^	Brazil	Analyze the monitoring of patients with hypertension during the pandemic by primary care.	Descriptive, qualitative study. A focus group technique was used with health professionals and managers.
Morais^(50)^	Brazil	Understanding barriers and facilitators to conducting consultations and measuring blood pressure.	Qualitative, descriptive study conducted with professionals from Basic Health Units.
Lopes^(51)^	Brazil	Understanding the impact of the care provided to people who experienced chronic illness during the COVID-19 pandemic, offered by the Hiperdia Program team.	Qualitative study, with a participatory approach, conducted with patients with hypertension and diabetes who were monitored remotely during the pandemic.

Source: Authors.

Captions: PHC: Primary Health Care; SAH: Systemic Arterial Hypertension.

Among the studies analyzed, most were municipal in scope (25 studies), followed by national studies (4 studies) and local studies (3 studies). Regarding the location of the research, despite the greater number of studies found in South American countries (Brazil, Chile, Colombia), with 17 publications, only five were scientific articles and 12 were theses and dissertations. The Asian continent stood out in the scientific production analyzed, with nine articles (Saudi Arabia, China, South Korea, India, Iran, Hong Kong, Tajikistan, Malaysia), followed by one produced in North America (Canada), one in Europe (Scotland), three in Oceania (Australia and Samoa), and one in Africa (South Africa).

### Effects of Continuity of Care on People with Systemic Arterial Hypertension

The effects of continuity of care on people with SAH, according to analytical categories emerging from the content analysis, are presented in Chart 2.

**Chart 2:** Summary of the effects of continuity of care for people with systemic arterial hypertension – Niterói, RJ, Brasil, 2025.

Analytic Category	Cause/effect
Blood Pressure Control	Patients who receive care from the same physician are more likely to achieve blood pressure control^(35,41)^.
	The number of consultations increases the chances of controlling blood pressure^(41)^.
Adherence to treatment	Regular monitoring of patients by the same professionals increases the chances of adherence to treatment^(11)^.
Reduction of target organ damage	Continuity of care is positively associated with a reduction in the overall risk of cardiovascular disease, coronary heart disease, and stroke^(42)^.
Use of health services and expenditures	Continuity of care is associated with a reduction in overall health costs/expenses^(12,13)^, a reduction in the frequency of hospitalization and use of hospital services (e.g., emergency rooms)^(14)^, fewer days of hospitalization, and lower hospitalization and outpatient costs^(15)^. Urban and rural areas have differences in hospitalization rates and use of services^(14).^
Quality of life	Patients treated by the same healthcare professionals experience better quality of life^(11)^.
Patient-professional communication	Healthcare providers with high levels of continuity of care are able to communicate better with their patients, understand their health history, and their current situation^(29)^.

Source: Authors.

### Limits in the Development of Continuity of Care for Patients with SAH in PHC

The limits of continuity of care for patients with SAH according to each dimension of continuity^(17)^ are systematized in Chart 3.

**Chart 3:** Factors that limit continuity of care for people with systemic arterial hypertension – Niterói, RJ, Brasil, 2025.

Continuity dimension	Factors limiting continuity
Management continuity	Lack of sharing of care strategies and the patient’s therapeutic plan among professionals at different levels of care, whether through referral and counter-referral or team meetings to discuss cases^(44,52,53)^.
	Lack of strategies for organizing supply/demand according to the clinical needs of patients with SAH^(9)^.
	Lack of risk categorization and blood pressure monitoring^(9,38)^.
	Long waiting times for care and insufficient consultation time with the professional^(9,27,33)^.
	Lack of agreement among health services in the region to ensure continuity of care for patients with SAH^(34)^.
	Insufficient availability of specialized tests and consultations for the diagnosis and management of SAH^(36)^.
	Lack of material resources for the implementation and continuity of treatment and monitoring of patients with SAH, especially antihypertensive drugs^(6,33,36,54,55)^.
	Lack of knowledge about the HAS care flow in the municipal network by PHC professionals^(27)^.
	Insufficient supply of equipment, such as sphygmomanometers, limits the ability to screen for SAH and monitor patients^(33,36,54)^.
Information continuity	Ineffective/non-existent communication between PHC professionals and specialized services^(9,52,55)^.
	Low integration among professionals at different levels of care within the network^(9,10)^.
	Care process centered on physicians and procedures, hindering communication and interaction among team members^(36,48,52)^.
	Failure to adequately fill out referral forms for patients with SAH for specialized care^(27)^.
	Lack of systematization and adequate storage of patient clinical data^(9,36)^. Incomplete and insufficient health records^(16)^.
Relational continuity	Turnover of PHC team professionals, hindering the formation of bonds^(31,52)^.
	Insufficient number of medical professionals in primary care given the growing demand from patients with hypertension^(9,27,52)^.
	Lack of user involvement in decision-making regarding their treatment plan^(36)^.
	Lack of commitment/accountability of Family Health teams to the patient’s therapeutic project^(34)^.

Source: Authors.

Captions: PHC: Primary Health Care; SAH: Systemic Arterial Hypertension.

### Strategies for Ensuring Continuity of Care for Patients with SAH

Although limited in number given the complexity of the topic, the strategies identified in the literature for ensuring continuity of care for patients with SAH were grouped according to the dimensions of care, as systematized in Chart 4. These strategies reflect initiatives aimed at organizing care, improving communication between services, and strengthening the bonds between users and professionals.

**Chart 4:** Strategies for ensuring continuity of care according to its dimensions – Niterói, RJ, Brasil, 2025.

Continuity dimension	Strategies
Informational continuity	Establishment of a referral and counter-referral system between levels of care^(29)^.
	Use of ICT, including remote care^(43)^.
	Adoption of standardized tools for assessing and monitoring continuity of care^(11)^.
Management continuity	Adoption of Chronic Care Model for organizing care for SAH^(36)^.
	Structuring formal care and organizational flows in PHC^(9)^.
	Health education for users, focusing on understanding hypertension and adherence to treatment^(38)^.
Relational continuity	Training of health professionals with a focus on continuous, patient-centered care^(44)^.
	Community engagement in reformulating health practices^(46)^.

Source: Authors.

Caption: ICT: Information and Communication Technologies.

## DISCUSSION

The results of this study highlight the importance of continuity of care^(56)^ for people diagnosed with SAH. Continuity of care not only improves the patient experience, but also has the potential to mitigate regional disparities in morbidity and mortality rates. This is especially relevant in urban and rural settings, where hospitalization rates related to SAH can vary considerably^(14)^.

Several limitations were identified in the continuity of care process, especially in the integration between different levels of care and resource management^(6,27,34,36,43,54)^. The absence of effective communication strategies^(9)^ and agreements between services and professionals, lack of knowledge about care flows^(33,38)^, and insufficient material and human resources^(27)^ compromise the quality of care for people with SAH.

In this sense, there is a need for communication strategies to strengthen the referral and counter-referral system and care flows, strengthen integration between services and care professionals, and implement integrated planning^(9)^. Linked to this, the low level of integration between PHC professionals and hospitals in developing strategies to control SAH highlights the lack of interprofessional collaboration^(30)^. The care process focused on the physician, in addition to hindering interaction and the approach to SAH treatment, often causes a heavy workload for medical professionals, hindering patient involvement in treatment decision-making and the division of tasks among team members^(34,52,55)^.

A care process offered by a multidisciplinary team can promote improved continuity of care through better coordination and articulation among its members during clinical practice^(42)^, in addition to promoting task sharing and division^(53)^.

Also noteworthy is the lack of systematization and organization of patient information in PHC, or even the incompleteness of clinical information. In many cases, professionals instruct users to manually record medication use, blood pressure monitoring, and other information important for follow-up, delegating responsibility for informational continuity to the individual themselves^(34)^.

A relevant strategy that can be adopted is the development of shared care plans between different levels of care, using information and communication technologies that facilitate the transition of care and the sharing of clinical information. Tools such as electronic medical records enable integrated access to the therapeutic project by professionals, promoting shared responsibility for care, better division of tasks within the team, and coordination between services^(27,46)^. For its application in the daily routine of services, it is recommended to institutionalize periodic meetings of multidisciplinary teams with time dedicated to the joint development of plans, definition of care goals, and case follow-up.

The Canadian experience with liaison nurses highlights the importance of professionals with specific responsibilities focused on the transition of care, such as planning hospital discharge and the safe transfer of clinical information between services. These nurses develop skills such as clinical judgment, leadership, and communication, acting as facilitators of continuity of care, especially in more complex cases, and supporting a practice centered on the needs and potential of patients. In turn, the experience in Portugal highlights the strengthening of interprofessional coordination and the focus on continuity of care through the implementation of individualized plans, developed with the participation of users and based on efficient communication between different levels of care^(57)^.

Furthermore, poor communication between health professionals and patients had direct implications for continuity, adherence to treatment, and control of SAH^(6)^. The goal should be to improve communication by adopting simpler language, without technical terminology, to facilitate understanding by users^(29)^.

The lack of risk categorization and adequate blood pressure monitoring^(43)^, coupled with long waiting times, reveals significant barriers to continuity and resolution of care, resulting in worsening of the disease, restricted access, difficulties in assessing treatment adherence, and lack of welcoming. These factors culminate in insecurity, discontinuity, and excessive financial costs for users and their families^(58)^.

These findings reflect broad difficulties in implementing the PHC-based care model, due to the value placed on the programmatic care model, which is still predominant in health services, with a focus on disease and treatment of acute conditions or exacerbation of chronic diseases, spontaneous demand, and curative and rehabilitative actions. In this context, the work of professionals focuses on disease, disability, and death^(59,60)^, thus hindering the continuity and coordination of care, comprehensive care, and health promotion.

Difficulties in accessing health services can limit the continuity of care for people with SAH, either due to geographical distances that make it difficult to attend appointments and have blood pressure measured^(9,43)^, or due to barriers encountered in undergoing tests and follow-up appointments^(9,38)^. In this scenario, it is essential to adopt strategies that optimize access and organization of the care schedule, especially with the use of digital technologies^(51)^. The incorporation of digital technologies, for example, has proven effective in the pandemic context and can be maintained as a complementary resource for monitoring people with SAH, especially in remote areas or areas with a shortage of professionals^(31)^. In addition, the use of online scheduling applications, sending automated appointment reminders to reduce absenteeism^(61)^, and expanding the offer of alternative service hours are measures that can be implemented in PHC practice, contributing to reducing access barriers and strengthening continuity of care.

In this context, it is worth highlighting the possibility of using the online scheduling system of the Electronic Citizens’Medical Record (PEC e-SUS APS in Portuguese), integrated with the apps”Conecte SUS Cidadão”, which allows patients to view available times, appointment schedules, and even cancellations without having to travel to the health center. This tool has the potential to reduce absenteeism, improve care schedule management, and expand access and resolution^(61)^.

A study^(62)^ showed that units with electronic medical records are more likely to function as the first point of contact and to meet urgent demands, demonstrating that computerization favors access and welcoming. Nevertheless, the adoption of these technologies requires attention to organizational and training aspects. Data indicate a growing use of telehealth and platforms such as the University Telemedicine Network (RUTE in the Portuguese acronym) and the Open University System of the Unified Health System (UNA-SUS), but also reveal that less than half of the health centers have adequate physical infrastructure for the effective use of these tools. This reinforces the need for investment in equipment, connectivity, and continuous training of professionals to successfully implement digital technologies in continuity of care strategies^(62)^. Thus, this approach can contribute to the longitudinal management of care, promote informational continuity, and strengthen coordination between points in the network.

There are also challenges related to the patient-professional relationship^(42,54)^, accountability of PHC teams^(43)^, and patient involvement in their therapeutic project^(27)^. These elements are intertwined, since the bond enables co-responsibility and continuity of care, constituting a fundamental strategy for strengthening the therapeutic relationship^(63)^.

The experience of organizing Health Care Networks in the eastern region of the Federal District, based on Health Care Planning, has shown that collaborative practices, qualified listening, and valuing user knowledge are necessary conditions for co-responsibility in care. This requires the development of skills beyond technical mastery, including the ability to dialogue, negotiate goals, and involve the patient in the decision-making process^(64)^.

Both the shortage and turnover of medical professionals in PHC have negative effects on establishing bonds with users and continuity of care^(48,52,63)^. On the other hand, hypertensive patients who remain under the care of the same physician for a period of two years have a significantly higher quality of life compared to those who receive care from multiple professionals. The bond between patient and professional not only promotes greater adherence to treatment but also increases the willingness to follow the guidelines provided^(11)^.

The findings also highlight that visits by patients to non-usual professionals can represent a loss of continuity and coordination of care, in addition to often increasing the number of referrals to specialized care, since these professionals are not familiar with the clinical history and have not developed a relationship of continuous care^(10)^.

Despite this, the high turnover of professionals, which is frequently observed, limits the quality and availability of care, constituting a barrier to access and weakening the professional-user bond^(65,66,67)^. Therefore, there is a need for policies that prioritize the stability of professionals in PHC and their retention in the territory.

The absence of a structured career path, with consolidated stability and functional progression, deepens professional turnover, weakens the bond with users, and disrupts the logic of the Health Care Networks in addition to reinforcing precarious and outsourced labor relations in health, with a strong impact on the discontinuity of care^(68)^.

In view of this, there is a reinforced need to strengthen PHC with structural investments that involve adequate financing, encouragement of intersectoral actions, valorization of health work, and implementation of policies to retain qualified professionals—such as the establishment of a public career in the SUS, a measure defended by social movements and researchers in the field of Health Reform as essential for the valorization of work with a view to consolidating a universal and quality SUS. Thus, a strong PHC is capable of ensuring continuity of care for its users^(68)^.

Against this backdrop, it can be observed that the effectiveness of continuity of care in PHC for users with SAH is directly related to the macro-political issues that guide the organization, functioning, and management of health systems^(69)^. In the Brazilian reality, the persistence of the hegemonic biomedical model—centered on procedures, disease, and acute conditions—disregards the epidemic of chronic conditions worldwide, and PHC reproduces the clinical practice of urgent and emergency care^(69,70)^. This scenario is aggravated by chronic underfunding and the defunding of the SUS imposed by Constitutional Amendment No. 95/2016^(70)^, which compromises the efficient allocation of resources and the sustainability of actions^(67)^. In addition, the training of health professionals, often guided by fragmented, mechanistic approaches to knowledge based on the positivist paradigm^(71)^, hinders the adoption and consolidation of interdisciplinary, person-centered, continuous, and coordinated practices in the health system. Furthermore, regional inequalities and the absence of effective policies to retain professionals in the most vulnerable regions amplify inequalities in access to quality services.

The ongoing training of PHC teams to develop continuous, person-centered care, based on guidelines aligned with the principles of the SUS, should promote the use of updated clinical protocols, but above all, the development of bonds and accountability. Such strategies are indispensable for overcoming the fragmentation of care and changing healthcare, contributing to the consolidation of comprehensive and coordinated practices and, consequently, promoting better health conditions for people with SAH.

The methodological heterogeneity of the included studies, which posed challenges for data analysis, stands out as a limitation of the study. On the other hand, the scarcity of studies with positive experiences of continuity implementation limited the identification of facilitators for the development of continuity of care.

## CONCLUSION

This study mapped the scientific production related to the care of people diagnosed with SAH, identifying effects, limitations, and strategies for the implementation of continuity of care in the context of PHC.

It revealed the importance of continuity of care in PHC for people with SAH, highlighting positive effects on blood pressure control, treatment adherence, reduction of morbidity and mortality, and decreased use of hospital services and health costs. Patients followed by the same professional are more likely to achieve blood pressure control and adhere to treatment, reducing the risk of cardiovascular disease and the frequency of hospitalizations. In addition, continuity of care improves the quality of life of hypertensive patients, as the bond with the same health professional facilitates adherence to treatment and acceptance of guidelines.

The mapped studies indicated the existence of important limitations to ensuring continuity of care in PHC. Among the limiting factors are the lack or discontinuity in the supply of medications, equipment for screening for SAH, such as sphygmomanometers, fragmentation of care, ineffective communication between professionals, and the absence of strategies for organizing care to meet patients’ clinical needs. The turnover of health professionals and the lack of patient involvement in therapeutic planning hinder the formation of bonds and the accountability of health teams.

Understanding these limitations and the strategies identified enables the recognition of weaknesses and the guidance of necessary adjustments to improve the continuity of care for people with SAH in PHC. In addition, it can support proposals for intervention in the territories, aiming at the implementation of strategies to address the identified challenges, as well as stimulate new investigations—especially longitudinal studies—that analyze the effects of the strategies adopted in continuity of care and explain successful experiences related to the practice of continuity of care in PHC.

## Data Availability

The entire data set supporting the results of this study was made available and published in the article itself.
