# Supplementary material for: Continuity of care for people with hypertension in primary health care: a scoping review
Source: Rev Esc Enferm USP. 2026 Jan 9;59:e20250053. doi: 10.1590/1980-220X-REEUSP-2025-0053en (PMC12788805; doi:10.1590/1980-220X-REEUSP-2025-0053en)
Supplement: Translation

# Translation: Continuidade do cuidado à pessoa com hipertensão na Atenção Primária à Saúde: uma revisão de escopo

**DOI:** 10.1590/1980-220X-REEUSP-2025-0053pt

**Authors:** Amanda Maria Villas Bôas Ribeiro, Patty Fidelis de Almeida, Ana Luiza Queiroz Vilasbôas

**Affiliations:** 1 Universidade Federal da Bahia, Instituto de Saúde Coletiva, Salvador, BA, Brasil.; 2 Universidade Federal Fluminense, Instituto de Saúde Coletiva, Niteroi, RJ, Brasil.

**Keywords:** Hipertensão, Continuidade da Assistência ao Paciente, Atenção Primária à Saúde

## Abstract

**Objetivo:**

Mapear as evidências sobre a continuidade do cuidado às pessoas com hipertensão arterial sistêmica na Atenção Primária à Saúde (APS), apontando limites e estratégias para a sua efetivação.

**Método:**

Revisão de escopo, sem delimitação temporal, a partir das bases de dados Web of Science, PubMed, Literatura Latino-Americana e do Caribe em Ciências da Saúde e a Scientific Electronic Library Online. Foram incluídas teses e dissertações da Biblioteca Digital Brasileira e do Catálogo da Coordenação de Aperfeiçoamento de Pessoal de Nível Superior. A seleção dos trabalhos foi realizada por duas revisoras independentes e às cegas, e as divergências resolvidas por uma terceira, totalizando corpus de 32 estudos. A extração dos dados foi realizada com base na adaptação do instrumento JBI, abrangendo informações sobre os estudos, metodologia e recomendações para a continuidade do cuidado. Os dados foram organizados em planilha Excel e analisados tematicamente, sendo interpretados à luz das dimensões da continuidade do cuidado.

**Resultados:**

A continuidade do cuidado em hipertensão na APS melhora o controle da pressão arterial, reduzindo morbidade, mortalidade e custos de saúde. Pacientes acompanhados pelo mesmo profissional de saúde apresentam melhor adesão ao tratamento e qualidade de vida. Foram identificados limites como a rotatividade de profissionais, fragilidade na comunicação entre serviços e a ausência de fluxos assistenciais consolidados. Como estratégias, destacam-se o fortalecimento do trabalho em equipe, a articulação interprofissional, o uso de prontuário eletrônico compartilhado e a adoção de fluxos formais de referência e contrarreferência.

**Conclusão:**

Implementar um cuidado baseado em equipe e promover o compartilhamento das atividades entre os profissionais são estratégias para alcançar a continuidade do cuidado.

## INTRODUÇÃO

Nas últimas três décadas, o número de pessoas com Hipertensão Arterial Sistêmica (HAS) no mundo passou de 650 milhões em 1990 para 1,3 bilhão em 2019, com estimativas de prevalência global de 49% entre homens e mulheres de 50 a 79 anos^(1)^, evidenciando sua magnitude. Diante da problemática, é fundamental assegurar a adesão à terapêutica medicamentosa e não-medicamentosa, mediante o acesso aos serviços de saúde, bom relacionamento com os profissionais^(2)^ e continuidade do cuidado para prevenção de riscos e agravos decorrentes da HAS, especialmente, a ocorrência de isquemia miocárdica, doença renal crônica e acidente vascular cerebral.

A Atenção Primária à Saúde (APS), de base territorial e orientação comunitária, apresenta um papel crucial no controle de doenças crônicas não transmissíveis, como no caso da HAS, devendo garantir acesso oportuno e adequado aos serviços da Rede de Atenção à Saúde (RAS), desempenhando a coordenação do cuidado, interligando recursos comunitários e assistenciais, para efetivação da continuidade do cuidado em saúde^(3)^.

Contudo, indivíduos com HAS enfrentam dificuldades de acesso aos serviços de saúde^(4,5)^, inclusive na APS, apesar de sua importância para controle da doença e diminuição de óbitos^(6–8)^. Os desafios vivenciados incluem desde o acesso restrito e fragmentado aos cuidados^(9)^, falta de recursos materiais e disponibilidade de medicamentos^(6)^, à ausência de comunicação entre os profissionais dos serviços de saúde^(10)^.

Diante destes entraves evidenciados, verifica-se a importância social da discussão ampliada acerca da continuidade do cuidado para criação de estratégias adequadas para sua efetivação na RAS sob coordenação da APS, uma vez que o acompanhamento regular e continuado do paciente com HAS diminui as chances de complicações graves e melhora a adesão terapêutica, autocuidado e qualidade de vida^(11)^, reduz a sobrecarga da média e alta complexidade com internações, exames e consultas^(12–15)^.

A continuidade do cuidado, muitas vezes, é confundida com longitudinalidade, coordenação ou mesmo com a integralidade da atenção^(16)^. Contudo, tais conceitos, apesar de relacionados, possuem diferenças. Considera-se a continuidade como oferta de cuidados integrados, coordenados e sequenciais ao longo do tempo, a partir do estabelecimento de vínculo e confiança, centrado em necessidades de saúde do paciente, com gerenciamento de informações clínicas compartilhadas por profissionais e serviços de saúde diferentes, sob coordenação da APS^(16,17)^. Vale salientar que a continuidade está atrelada à experiência individual de cada usuário no transcurso de seu projeto terapêutico relacionado a um problema de saúde específico^(18)^, como a HAS.

O objetivo do artigo é mapear as evidências sobre o desenvolvimento da continuidade do cuidado às pessoas com diagnóstico de HAS pela APS, apontando limites e estratégias para a sua efetivação. Foi realizada busca preliminar com os Medical SubjectHeadings (MeSH) “carecontinuity” e “hypertension” no Open Science Framework, Joanna Briggs Institute Evidence Synthesis não sendo encontrados estudos ou protocolos de revisão de escopo sobre a temática, o que justifica a relevância do artigo.

## MÉTODO

### Desenho do Estudo

Trata-se de uma revisão de escopo realizada conforme o método recomendado pelo *Joanna Briggs Institute* (JBI)^(19)^ e apresentada segundo recomendações do *checklist Preferred ReportingItems for Systematic reviews and Meta-Analysesextension for Scoping Reviews* (PRISMA-ScR)^(20)^.

Este tipo de pesquisa consiste em uma revisão exploratória destinada a mapear a literatura relevante na área de interesse e os seus conceitos-chave, bem como identificar lacunas na literatura científica que auxiliem na definição de questões de investigação^(21)^. As etapas da revisão de escopo incluem: formulação da questão e objetivo da pesquisa; identificação de documentos relevantes que estejam de acordo com a amplitude e abrangência dos propósitos da revisão; seleção dos estudos conforme os critérios predefinidos; mapeamento de dados; sumarização dos resultados, por meio de análise temática qualitativa em relação ao objetivo e pergunta; e apresentação dos resultados^(19)^.

O protocolo desta revisão, delineado a partir da questão de pesquisa, foi registrado na plataforma para registro de trabalhos científicos Open Science Framework por meio do link osf.io/tdr6y.

### Pergunta Norteadora

Delineou-se a questão de pesquisa com base na estratégia mnemônica Problema, Conceito e Contexto (PCC). Tal metodologia auxilia na identificação dos tópicos chaves para nortear a coleta de dados.

No que tange aos Participantes (P) foram definidos como pessoas com diagnóstico de HAS. No que diz respeito ao Conceito (C) foram incluídos nesta revisão estudos que abordem a continuidade do cuidado à pessoa com Hipertensão Arterial Sistêmica (HAS). O conceito de continuidade do cuidado envolve a relação estabelecida entre o profissional de saúde e o paciente que se prolonga para além dos episódios de adoecimento, sendo considerado um “reflexo de um senso de afiliação, frequentemente expresso em termos de contrato implícito de lealdade por parte do paciente e responsabilidade clínica por parte do médico”, tendo como pré-requisito o acesso aos serviços de saúde e a coordenação do cuidado como agente facilitador^(17)^. E, por sua vez, no que concerne ao Contexto (C) foram selecionados estudos realizados na Atenção Primária à Saúde, com abrangências nacionais, regionais, municipais, distritais ou locais.

Desse modo, conciliando os tópicos chaves do PCC e o objetivo do estudo, tem-se como pergunta de investigação: Como se desenvolve a continuidade do cuidado às pessoas com diagnóstico de HAS pela APS?

### Estratégias de Busca

Esta revisão foi realizada nas seguintes bases de dados: *Web of Science Core Collection, PubMed* via *National Library of Medicine/The National Center for BiotechnologyInformation* (NLM/NCBI), Literatura Latino-Americana e do Caribe em Ciências da Saúde (LILACS) via Biblioteca Virtual em Saúde e a *ScientificElectronic Library Online* (SciELO). A busca da literatura cinzenta foi executada através da Biblioteca Digital Brasileira de Teses e Dissertações e Catálogo CAPES.

Foram utilizados termos livres associados aos descritores do *Medical SubjectHeadings* (MeSH) e os Descritores em Ciências da Saúde (DeCS): *Primary Health Care, Hypertension* e *Continuityofpatientcare*. As estratégias de busca seguiram as definições da base de dados correspondente e foram desenvolvidas com apoio de um bibliotecário especializado em revisões. A coleta de dados foi realizada em janeiro de 2024.

### Critérios de Elegibilidade

Conforme recomendações^(22)^, foram definidos critérios de inclusão e exclusão para seleção dos artigos. Assim, as fontes de evidências desta revisão foram textos completos de estudos primários, teses e dissertações, todos publicados em bases de dados da área da saúde. Foram incluídos estudos que abordassem a continuidade do cuidado à pessoa com HAS, com documentos disponíveis na íntegra, publicados em português, espanhol ou inglês, sem limites temporais definidos, com intuito de recuperar o maior número possível de publicações relacionadas à questão de pesquisa. Não foram incluídos artigos de revisão, protocolos de estudos de revisão, artigos de opinião, anais de congresso, comentários, editoriais, capítulos de livros.

### Extração dos Dados

Os artigos encontrados na busca foram agrupados e importados para a ferramenta *RayyanIntelligentSystematic Review* para exclusão de duplicatas e seleção dos trabalhos. Em seguida, realizou-se de forma independente e às cegas a seleção das fontes de evidências por duas revisoras a partir da leitura dos títulos e resumos. As divergências encontradas foram resolvidas por uma terceira revisora e os motivos para exclusão dos manuscritos foram justificados. Posteriormente, os estudos que atenderam aos critérios foram avaliados na íntegra para uma completa apreciação do material retido e composição da amostra final para a extração dos dados.

A extração dos dados foi organizada a partir da adaptação do instrumento desenvolvido pelo JBI^(20)^ para atender aos objetivos deste estudo, contendo informações a respeito da identificação dos autores, título completo, ano de publicação, objetivos, metodologia (tipo de estudo, métodos adotados, participantes e/ou fontes de dados, cenário, abrangência), síntese dos resultados, dificuldades e facilidades para desenvolvimento da continuidade do cuidado a pessoas com HAS no âmbito da APS e recomendações do estudo para alcance da continuidade do cuidado. Todas as etapas da extração dos dados dos estudos selecionados foram feitas com uso de planilha *Excel*.

### Análise e Apresentação dos Dados

Para descrição dos resultados da busca e seleção dos estudos, foi utilizado o fluxograma de identificação, escaneamento e processo de inclusão adaptado do *PreferredReportingItems for Systematic Review and Meta-analyses* (PRISMA-ScR)^(19)^.

Após a seleção dos estudos, os dados extraídos foram organizados em um instrumento de coleta adaptado^(20)^, contendo informações como autores, ano, país, objetivos, desenho metodológico, principais achados e aspectos relacionados à continuidade do cuidado. A análise dos dados foi conduzida por meio da análise temática de conteúdo^(23)^, aplicada de forma sistemática aos dados qualitativos e quantitativos.

Nos estudos quantitativos, os achados numéricos foram interpretados à luz de seus significados e implicações descritas nos próprios artigos, sendo categorizados de acordo com as dimensões temáticas emergentes, tais como barreiras, efeitos e estratégias associadas à continuidade do cuidado. Assim, os dados quantitativos não foram analisados estatisticamente, mas integrados à análise qualitativa com base em seu conteúdo interpretativo, conforme sua relevância para o objeto deste estudo.

O processo de análise seguiu as seguintes etapas: (1) leitura flutuante dos textos completos; (2) codificação dos dados extraídos no instrumento de coleta; (3) categorização dos conteúdos por similaridade temática; (4) identificação de temas centrais relacionados ao objeto da revisão, especialmente os que tratavam dos limites, efeitos e estratégias para efetivação da continuidade do cuidado.

Os resultados da análise foram organizados e apresentados em quadros explicativos, com sínteses narrativas, e interpretados à luz das dimensões da continuidade do cuidado^(17)^. A continuidade do cuidado pode ser compreendida a partir de três dimensões principais: gerencial, informacional e relacional. A continuidade gerencial refere-se à gestão do cuidado ao longo do tempo e dos diferentes pontos da rede de atenção à saúde, exigindo a articulação entre serviços e a gestão clínica adequada às necessidades do usuário^(17,18)^. A continuidade informacional possibilita o compartilhamento efetivo de informações relevantes sobre o histórico, o contexto e os cuidados de saúde do usuário entre os profissionais e os serviços^(16,17)^. Por fim, a continuidade relacional envolve o estabelecimento de vínculos terapêuticos duradouros entre usuários e profissionais de saúde, sustentando a confiança, o acolhimento e a percepção de cuidado contínuo ao longo do tempo^(17,18)^.

### Aspectos Éticos

Os dados utilizados possuem acesso público, deste modo, não houve necessidade de submissão ao Comitê de Ética em Pesquisa.

## RESULTADOS

Inicialmente, foram identificados 230 estudos, sendo 214 artigos nas bases de dados e 16 dissertações e teses. Destes estudos, 75 eram duplicados e por isso foram removidos. Após leitura dos títulos e resumos realizada por duas pesquisadoras de modo duplo cego, 89 estudos foram excluídos, por não tratarem da temática ou não atenderem aos critérios estabelecidos. Procedeu-se à leitura na íntegra de 66 estudos, sendo excluídos 34, dois por não estarem disponíveis na íntegra, sete pelo tipo de publicação (anais, protocolos de estudos e diretrizes terapêuticas), 22 por não responderem à pergunta de pesquisa, dois não tratavam da população em questão, e um não tinha relação com o conceito de continuidade do cuidado.

Assim, a amostra final foi constituída por 32 estudos, sendo 20 artigos científicos e 12 teses e dissertações. No intuito de sistematizar o processo de seleção dos estudos, utilizou-se o PRISMA-SrC^(20)^ (Figura 1).

**Figura 1:** Fluxograma do processo de seleção dos estudos sobre continuidade do cuidado à pessoa com HAS no contexto da Atenção Primária à Saúde.

A seguir, buscou-se apresentar a caracterização dos estudos analisados e a síntese dos achados a partir de duas categorias abrangentes identificadas a partir da análise de conteúdo: efeitos da continuidade no cuidado em saúde das pessoas com HAS e desafios no desenvolvimento da continuidade do cuidado ao paciente com HAS na APS.

### Caracterização dos Estudos

A dimensão temporal dos estudos correspondeu ao período entre 2005 e 2023, conforme Quadro 1. Observa-se um incremento gradativo de publicações a partir de 2019, com cerca de 52,94% dos estudos publicados entre os anos de 2019 e 2023. Houve predominância de estudos quantitativos (18 trabalhos), especialmente transversais (10 trabalhos).

**Quadro 1:** Caracterização dos estudos selecionados segundo objetivos, metodologia, idioma e ano de publicação – Niterói, RJ, Brasil, 2025.

Autores	País	Objetivo	Metodologia
Inkster et al.^(24)^	Escócia	Investigar aspectos do manejo da hipertensão em relação às variáveis sociodemográficas, tratamento medicamentoso anti-hipertensivo e fatores organizacionais na atenção primária.	Quantitativo, observacional, realizado com 560 pacientes selecionados aleatoriamente com idades entre 40 e 79 anos em tratamento para hipertensão. Foram coletados dados relativos ao processo de cuidado.
Cunha^(25)^	Brasil	Investigar o atendimento ao atributo do vínculo longitudinal na experimentação de diferentes modelos assistenciais organizativos da APS no contexto do SUS.	Estudo qualitativo realizado por meio de entrevista semiestruturada com os profissionais, revisão de amostra de prontuários e aplicação de inquérito em amostra de usuários.
Malfatti e Assunção^(8)^	Brasil	Avaliar a média anual do cadastro de diabetes e hipertensão e o correspondente nível de acompanhamento dos pacientes pelos municípios.	Estudo transversal, com uso de registros de diabetes e hipertensão e o acompanhamento dos profissionais de saúde das equipes de APS dos municípios de uma região de saúde do Rio Grande do Sul.
Machado^(26)^	Brasil	Avaliar o grau de integração da rede de serviços de saúde no atendimento às pessoas com HAS.	Estudo avaliativo, realizado com gestores e equipe técnica da atenção primária, vigilâncias, cardiologia, urgência e emergência, atenção psicossocial, rede hospitalar e regulação.
Martins^(27)^	Brasil	Analisar o acesso e a continuidade do cuidado dos pacientes com HAS e Diabetes Mellitus.	Estudo misto, com análise documental, inquérito telefônico e entrevista semiestruturada. Realizou-se análise comparativa segundo o tipo de unidade de saúde: Unidades do Programa Médico de Família, Unidades Básicas e Policlínicas.
Sousa et al.^(9)^	Brasil	Analisar as condições para o acesso integral na rede assistencial considerando o atendimento em uma Unidade de Saúde da Família até a assistência especializada.	Estudo de métodos mistos, com uso de grupos focais com profissionais da APS e coleta de dados do Sistema da Central de Regulação da Saúde e do Sistema de Marcação de Consultas.
Katz et al.^(10)^	Canadá	Analisar modos de acesso aos cuidados de saúde de adultos com doenças crônicas.	Coorte realizada com 347.606 pacientes com pelo menos quatro consultas ambulatoriais com médico de atenção primária ou especialista no período do estudo.
Hanafi et al.^(28)^	Malásia	Investigar a relação entre continuidade pessoal e controle da pressão arterial entre pacientes com hipertensão em um centro acadêmico de atenção primária.	Estudo de coorte longitudinal retrospectivo realizado através da revisão de 1.060 prontuários de pacientes adultos com hipertensão.
Li et al.^(29)^	China	Comparar qualidade percebida da atenção primária em termos de acessibilidade, continuidade, coordenação e integralidade entre pacientes hipertensos.	Estudo transversal, com uso da versão chinesa adaptada e validada do *Primary Care Assessment Tool* (PCAT).
Li et al.^(30)^	China	Comparar a continuidade do cuidado entre pacientes hipertensos entre o Modelo de Gestão Direta de centros de saúde comunitários e o Modelo de Colaboração Loose.	Estudo transversal realizado em 4 Centros de saúde da China aleatoriamente, com 386 pacientes em Nanjing e 396 em Wuhan.
Baricati^(31)^	Brasil	Compreender a longitudinalidade do cuidado a partir das experiências dos usuários da APS.	Estudo qualitativo, do tipo descritivo e exploratório, realizado na zona urbana de um município.
Li et al.^(32)^	China	Investigar as mudanças na qualidade percebida dos cuidados primários por pacientes idosos hipertensos.	Pesquisa transversal realizada em duas fases com pacientes atendidos na Atenção Primária à Saúde de Xangai.
Wollum et al.^(33)^	África do Sul	Identificar características básicas e barreiras ao tratamento da hipertensão, hipercolesterolemia e diabetes.	Estudo de métodos mistos com inquéritos nas unidades da APS discussões em grupos focais e entrevistas com informadores-chave.
Santos^(34)^	Brasil	Avaliar a articulação entre serviços de atenção primária e atenção especializada no cuidado à pacientes hipertensos.	Estudo quanti-qualitativo realizado com médicos da atenção primária à saúde.
Khanam et al.^(35)^	Austrália	Verificar a relação entre o controle da Pressão Arterial e a continuidade do cuidado.	Estudo quantitativo, dados coletados de 329 clínicas gerais localizadas em toda a Austrália.
Lall et al.^(36)^	Índia	Compreender o modo de organização dos cuidados primários para a diabetes e a hipertensão nas unidades de saúde da Índia.	Estudo de métodos mistos com entrevistas em profundidade 24 pacientes com diabetes, hipertensão e 6 médicos em três Unidades de saúde e três unidades privadas.
Silva^(37)^	Brasil	Avaliar o efeito do gerenciamento de caso na pressão arterial e nas complicações em adultos com hipertensão.	Ensaio clínico randomizado realizado em uma unidade de saúde da atenção primária.
Chukwuma et al.^(38)^	Tajikistão	Compreender as lacunas na continuidade dos cuidados na APS, desafios e propor soluções.	Estudo de métodos mistos. Grupos focais com usuários de serviços, prestadores de cuidados de saúde e administradores.
Leniz e Gulliford^(39)^	Chile	Explorar fatores relacionados à continuidade dos cuidados e sua associação com cuidados com diabetes e hipertensão e controle da doença.	Estudo transversal que baseou-se em dados da Pesquisa Nacional de Saúde realizada no Chile com usuários da APS.
Neves^(40)^	Brasil	Analisar como se configuram as relações entre os profissionais da Estratégia de Saúde da Família quanto à assistência aos usuários com hipertensão e diabetes.	Estudo descritivo, com abordagem qualitativa e quantitativa.
Barrera et al.^(41)^	Colômbia	Identificar as condições associadas ao alcance da hipertensão arterial controlada.	Estudo observacional longitudinal com indivíduos diagnosticados com hipertensão arterial atendidos em dois centros de APS.
Fraser-Hurt et al.^(6)^	Samoa	Avaliar problemas na continuidade do cuidado e identificar possíveis soluções, utilizando a hipertensão como condição traçadora.	Estudo de métodos mistos, com utilização de dados de inquéritos domiciliares de adultos integrados com dados de prontuários médicos de pacientes com hipertensão e resultados de discussões de grupos focais com pacientes e profissionais de saúde.
Xu et al.^(42)^	Hong Kong	Investigar a eficácia da continuidade do cuidado baseado em equipe na prevenção de doenças cardiovasculares e mortalidade em pacientes com hipertensão.	Coorte retrospectiva realizada com pacientes que visitaram a atenção primária em Hong Kong de 2008 a 2018.
Bellas et al.^(43)^	Brasil	Avaliar o desempenho resiliente quanto à continuidade da atenção e ao cuidado longitudinal à hipertensão arterial sistêmica e diabetes Mellitus pelas unidades de atenção básica durante a pandemia da Covid-19.	Estudo de métodos mistos, de abordagem quantitativa e qualitativa. Utilizou-se dados secundários do Sistema de Informação e entrevistas em profundidade com gestores e profissionais técnicos de gestão da coordenadoria de área programática do município do Rio de Janeiro-Brasil.
Almalki et al.^(44)^	Arábia Saudita	Explorar a continuidade do cuidado e seus preditores em ambientes de Atenção Primária à Saúde em pacientes com doenças crônicas na Arábia Saudita.	Estudo transversal, realizado com 193 usuários da APS com doenças crônicas que tiveram pelo menos quatro visitas anteriores às unidades básicas de saúde no último ano.
Trivedi et al.^(45)^	Austrália	Explorar fatores que podem influenciar o controle da pressão arterial em pacientes com fibrilação atrial e com hipertensão.	Estudo retrospectivo, transversal. Utilizou-se um banco de dados que inclui registros eletrônicos de saúde de clínicas gerais.
Dehghani Tafti et al.^(46)^	Irã	Determinar a continuidade do cuidado de pacientes com condições crônicas como hipertensão e diabetes durante a pandemia de COVID-19.	Estudo retrospectivo transversal. A população do estudo consistiu em todos os pacientes que sofrem de diabetes tipo 2 e hipertensão e que tinham um prontuário médico ativo em um dos centros de saúde de há pelo menos dois anos antes da pandemia de COVID-19.
Damião^(47)^	Brasil	Analisar o impacto da pandemia na continuidade do cuidado de pacientes com HAS e Diabetes Mellitus no município de Pinhais-PR.	Estudo longitudinal, retrospectivo, com 540 pacientes com Hipertensão ou Diabetes, acompanhados por Unidades de Saúde da Família.
Cardoso^(48)^	Brasil	Analisar a priorização dos atendimentos no combate a COVID-19 em relação aos atendimentos sistemáticos de pacientes hipertensos, diabéticos, gestantes e crianças na Atenção Primária do Distrito Federal.	Estudo transversal, com análise de dados de pacientes hipertensos, diabéticos, gestantes e crianças acompanhadas na APS do período de 2018 a 2020, no Distrito Federal.
Cavalcante^(49)^	Brasil	Analisar o acompanhamento de pacientes com hipertensão durante a pandemia pela atenção primária.	Estudo descritivo, qualitativo. Utilizou-se técnica de grupo focal com profissionais de saúde e gestores.
Morais^(50)^	Brasil	Compreender barreiras e facilitadores da realização de consultas e aferição de pressão arterial.	Estudo qualitativo, descritivo, realizado com profissionais de Unidades Básicas de Saúde.
Lopes^(51)^	Brasil	Compreender as repercussões do cuidado oferecido às pessoas que vivenciaram o adoecimento crônico durante a pandemia por covid-19, oferecido pela equipe do Programa Hiperdia.	Estudo qualitativo, de abordagem participativa, realizado com pacientes com Hipertensão e Diabetes e em acompanhamento remoto durantea pandemia.

Fonte: Construção própria. Legenda: APS: Atenção Primária à Saúde; HAS: Hipertensão Arterial Sistêmica.

Dentre os estudos analisados, a maioria possuía abrangência municipal (25 estudos), seguido por estudos nacionais (4 estudos) e locais (3 estudos). No que tange ao local de realização das pesquisas, apesar do maior quantitativo de estudos encontrados em países da América do Sul (Brasil, Chile, Colômbia), com 17 produções, apenas cinco eram artigos científicos e 12 teses e dissertações. O continente asiático destacou-se na produção científica analisada, com 9 artigos (Arábia Saudita, China, Coreia do Sul, Índia, Irã, Hong Kong, Tajiquistão, Malásia), seguido de um elaborado na América do Norte (Canadá), um na Europa (Escócia), três na Oceania (Austrália e Samoa), e um no continente africano (África do Sul).

### Efeitos da Continuidade no Cuidado em Saúde das Pessoas com Hipertensão Arterial Sistêmica

Os efeitos da continuidade do cuidado em saúde das pessoas com HAS, segundo categorias analíticas emergidas da análise de conteúdo, são apresentados no Quadro 2.

**Quadro 2:** Síntese dos efeitos da continuidade do cuidado da pessoa com hipertensão arterial sistêmica – Niterói, RJ, Brasil, 2025

Categoria analítica	Causa/efeito
Controle da Pressão Arterial	Pacientes que recebem cuidado do mesmo médico possuem maior probabilidade de alcançar controle da pressão arterial^(35,41)^.
	O número de consultas realizadas aumenta as chances de controle da pressão arterial^(41)^.
Adesão ao tratamento	O acompanhamento regular dos pacientes pelos mesmos profissionais aumenta as chances de adesão ao tratamento^(11)^.
Redução de lesões em órgãos-alvo	A continuidade do cuidado associa-se positivamente à redução do risco global da ocorrência de doenças cardiovasculares, doença coronariana e Acidente Vascular Cerebral^(42)^.
Uso de serviços e gastos com saúde	A continuidade do cuidado associa-se à redução dos custos/gastos globais com saúde^(12,13)^ redução da frequência de hospitalização e de uso dos serviços hospitalares (pronto-socorro, por exemplo)^(14)^, menos dias de internação e diminuição de custos de internação e ambulatório^(15)^. Áreas urbanas e rurais têm diferenças nas taxas de hospitalização e uso de serviços^(14)^.
Qualidade de vida	Pacientes tratados pelos mesmos profissionais de saúde experimentam melhores níveis de qualidade de vida^(11)^.
Comunicação paciente-profissional	Prestadores de cuidados com elevados níveis de continuidade do cuidado são capazes de se comunicar melhor com seus pacientes, conhecer o histórico de saúde do paciente e sua situação atual^(29)^.

Fonte: construção própria.

### Limites no Desenvolvimento da Continuidade do Cuidado ao Paciente com HAS na APS

Os limites da continuidade do cuidado ao paciente com HAS segundo cada dimensão da continuidade^(17)^ estão sistematizados no Quadro 3.

**Quadro 3:** Fatores que limitam a continuidade do cuidado à pessoa com hipertensão arterial sistêmica – Niterói, RJ, Brasil, 2025.

Dimensão da continuidade	Fatores limitantes da continuidade
Continuidade gerencial	Ausência de compartilhamento das estratégias de cuidado e do projeto terapêutico do paciente entre os profissionais de diferentes níveis de atenção, seja por meio de referência e contrarreferência ou reuniões entre equipes para discussão dos casos^(44,52,53)^.
	Ausência de estratégias para organização da oferta/demanda segundo critérios de necessidades clínicas dos pacientes com HAS^(9)^.
	Ausência de categorização de risco e de monitoramento da pressão arterial^(9,38)^.
	Longos tempos de espera para atendimento e tempo insuficiente de consulta com o profissional^(9,27,33)^.
	Ausência de pactuação dos serviços de saúde do território para garantia do fluxo assistencial do paciente com HAS^(34)^.
	Oferta insuficiente de exames e consultas especializadas para diagnóstico e controle da HAS^(36)^.
	Falta de recursos materiais para implementação e continuidade do tratamento e monitoramento do paciente com HAS, especialmente medicamentos anti-hipertensivos^(6,33,36,54,55)^.
	Desconhecimento do fluxo assistencial da HAS na rede municipal pelos profissionais da APS^(27)^.
	Oferta insuficiente de equipamentos, como esfigmomanômetro, limitam a capacidade de rastreamento da HAS e do monitoramento^(33,36,54)^.
Continuidade informacional	Comunicação ineficaz/inexistente entre os profissionais da APS e serviços especializados^(9,52,55)^.
	Baixa integração entre os profissionais dos distintos níveis de atenção da rede^(9,10)^.
	Processo de cuidado centrado no médico e procedimentos, dificultando comunicação e interação entre membros da equipe^(36,48,52)^.
	Falta de preenchimento adequado das fichas de referência dos pacientes com HAS para atenção especializada^(27)^.
	Ausência de sistematização e armazenamento adequado dos dados clínicos dos pacientes^(9,36)^. Incompletude e insuficiência dos registros em saúde^(16)^.
Continuidade relacional	Rotatividade dos profissionais da equipe da APS, prejudicando a formação de vínculo^(31,52)^.
	Insuficiente quantitativo de profissionais médicos na APS diante da crescente demanda de pacientes com HAS^(9,27,52)^.
	Falta de envolvimento dos usuários na tomada de decisão do seu projeto terapêutico^(36)^.
	Falta de comprometimento/responsabilização das equipes de Saúde da Família com o projeto terapêutico do paciente^(34)^.

Fonte: Construção própria. Legenda: APS: Atenção Primária à Saúde; HAS: Hipertensão Arterial Sistêmica.

### Estratégias Para a Efetivação da Continuidade do Cuidado ao Paciente com HAS

Embora em quantidade limitada frente à complexidade do tema, as estratégias identificadas na literatura para a efetivação da continuidade do cuidado ao paciente com HAS foram agrupadas segundo as dimensões do cuidado, conforme sistematizado no Quadro 4. Tais estratégias refletem iniciativas voltadas à organização do cuidado, à qualificação da comunicação entre os serviços e ao fortalecimento dos vínculos entre usuários e profissionais.

**Quadro 4:** Estratégias para efetivação da continuidade do cuidado segundo suas dimensões – Niterói, RJ, Brasil, 2025.

Dimensão da continuidade	Estratégias
Continuidade Informacional	Estabelecimento de sistema de referência e contra-referência entre os níveis de atenção^(29)^.
	Uso de TIC, incluindo atendimentos remotos^(43)^.
	Adoção de ferramentas padronizadas de avaliação e monitoramento da continuidade do cuidado^(11)^.
Continuidade Gerencial	Adoção do MACC para organização da atenção à HAS^(36)^.
	Estruturação de fluxos assistenciais e organizacionais formais na APS^(9)^.
	Educação em saúde para usuários, com foco na compreensão da hipertensão e adesão ao tratamento^(38)^.
Continuidade relacional	Formação de profissionais de saúde com foco em cuidados contínuos e centrados no paciente^(44)^.
	Engajamento comunitário na reformulação de práticas em saúde^(46)^.

Fonte: Construção própria. Legenda: MACC: Modelo de Cuidados Crônicos; TIC: Tecnologias da Informação e Comunicação.

## DISCUSSÃO

Os resultados deste estudo sinalizam a importância da continuidade do cuidado^(56)^ para as pessoas com diagnóstico de HAS. A continuidade do cuidado não só melhora a experiência do paciente, mas também possui o potencial de mitigar disparidades regionais nas taxas de morbimortalidade. Isso é especialmente relevante em contextos urbanos e rurais, onde as taxas de hospitalização relacionadas à HAS podem variar consideravelmente^(14)^.

Foram identificadas diversas limitações no processo de continuidade do cuidado, especialmente na integração entre os diferentes níveis de atenção e a gestão de recursos^(6,27,34,36,43,54)^. A ausência de estratégias eficazes de comunicação^(9)^ e pactuação entre os serviços e profissionais, desconhecimento dos fluxos assistenciais^(33,38)^, e a insuficiência de recursos materiais e humanos^(27)^ comprometem a qualidade do cuidado às pessoas com HAS.

Nesse sentido, aponta-se a necessidade de estratégias comunicacionais para fortalecimento do sistema de referência e contrarreferência e fluxos assistenciais, o fortalecimento da integração entre serviços e profissionais da assistência e implementação de um planejamento integrado^(9)^. Atrelado a isso, a baixa integração entre profissionais da APS e hospitais para construção de estratégias de controle da HAS evidencia a ausência de colaboração interprofissional^(30)^. O processo de cuidado concentrado na figura do médico, além de dificultar a interação e abordagem do tratamento da HAS, provoca frequentemente grande sobrecarga de atendimento aos profissionais médicos, dificultando o envolvimento do paciente na tomada de decisão acerca do tratamento e a divisão de tarefas entre membros da equipe(34,52,55).

Um processo de cuidado ofertado por equipe multiprofissional pode promover a melhoria da continuidade do cuidado, por meio de melhor coordenação e articulação entre seus membros durante a prática clínica^(42)^, além de promover compartilhamento e divisão de tarefas^(53)^.

Destaca-se ainda a falta de sistematização e organização das informações sobre os pacientes na APS, ou até mesmo incompletude das informações clínicas. Em muitos casos, os profissionais orientam os usuários a registrar manualmente o uso de medicamentos, monitoramento da PA e outras informações importantes ao acompanhamento, delegando ao próprio indivíduo a responsabilidade pela continuidade informacional^(34)^.

Uma estratégia relevante que pode ser adotada é a elaboração de planos de cuidado compartilhados entre os diferentes níveis de atenção, com uso de tecnologias de informação e comunicação que facilitem a transição do cuidado e o compartilhamento de informações clínicas. Ferramentas como os prontuários eletrônicos possibilitam o acesso integrado ao projeto terapêutico por parte dos profissionais, favorecendo a corresponsabilização pelo cuidado, melhor divisão de tarefas na equipe e articulação entre os serviços^(27,46)^. Para sua aplicação no cotidiano dos serviços, recomenda-se a institucionalização de reuniões periódicas das equipes multiprofissionais com momentos dedicados à construção conjunta dos planos, definição das metas assistenciais e acompanhamento dos casos.

A experiência canadense com as enfermeiras de ligação, evidencia a importância de profissionais com atribuições específicas voltadas à transição do cuidado, como o planejamento da alta hospitalar e a transferência segura de informações clínicas entre serviços. Essas enfermeiras desenvolvem competências como julgamento clínico, liderança e comunicação, atuando como facilitadoras da continuidade do cuidado, sobretudo nos casos mais complexos, e apoiando uma prática centrada nas necessidades e potencialidades dos pacientes. Por sua vez, a experiência de Portugal destaca o fortalecimento da articulação interprofissional e o foco na continuidade do cuidadopor meio da implementação de planos individualizados, construídos com a participação dos usuários e baseados na comunicação eficiente entre os diferentes níveis de atenção^(57)^.

Ademais, a frágil comunicação entre profissionais de saúde e pacientes apresentou implicações diretas à continuidade, adesão ao tratamento e controle da HAS^(6)^. Deve-se almejar a melhoria da comunicação com adoção de linguagem mais simples, sem terminologias técnicas para facilitar a compreensão por parte dos usuários^(29)^.

A inexistência de categorização de risco e monitoramento adequado da pressão arterial^(43)^ atrelada aos longos tempos de espera revela barreiras significativas para a continuidade e resolutividade do cuidado, reverberando em agravamento da doença, restrição do acesso, dificuldades na avaliação da adesão ao tratamento e ausência de acolhimento. Estes fatores culminam em insegurança, descontinuidade e gastos financeiros excessivos para usuários e suas famílias^(58)^.

Tais achados refletem dificuldades amplas na implementação do modelo assistencial baseado na APS, em virtude da valorização do modelo de atenção programática, ainda predominante nos serviços de saúde, com foco na doença e tratamento de condições agudas ou agudização de doenças crônicas, na demanda espontânea e em ações curativistas reabilitadoras. Neste contexto, o trabalho dos profissionais incide sobre a doença, a incapacidade e o óbito^(59,60)^ e, desse modo, dificulta a continuidade e coordenação do cuidado, atenção integral e promoção de saúde.

Dificuldades de acesso aos serviços de saúde podem limitar a continuidade do cuidado à pessoa com HAS, seja pelas distâncias geográficas que dificultam a realização de consultas e aferição da pressão arterial^(9,43)^, seja pelas barreiras encontradaspara realização de exames e consultas para seguimento^(9,38)^. Neste cenário, é fundamental adotar estratégias que otimizem o acesso e organização da agenda assistencial, principalmente com uso das tecnologias digitais^(51)^. A incorporação das tecnologias digitais, por exemplo, mostrou-se eficaz no contexto pandêmico e pode ser mantida como recurso complementar para o acompanhamento de pessoas com HAS, especialmente em áreas remotas ou com escassez de profissionais^(31)^. Além disso, o uso de aplicativos de agendamento online, envio de lembretes automatizados de consultas para redução do absenteísmo^(61)^, e a ampliação da oferta de horários alternativos de atendimento são medidas que podem ser implementadas na prática da APS, contribuindo para reduzir barreiras de acesso e fortalecer a continuidade do cuidado.

Destaca-se, nesse contexto, a possibilidade de uso do sistema de agendamento online do Prontuário Eletrônico do Cidadão (PEC e-SUS APS), integrado ao aplicativo conecte SUS cidadão, que permite aos pacientes a visualização dos horários disponíveis, agendas de consultas e até cancelamentos sem a necessidade de deslocamento até a UBS. Esta ferramenta tem potencial para redução do absenteísmo, melhoria da gestão da agenda assistencial e ampliação do acesso e resolutividade^(61)^.

Um estudo^(62)^ demonstrou que unidades com prontuário eletrônico têm maior chance de funcionar como primeiro ponto de contato e de atender demandas urgentes, evidenciando que a informatização favorece o acesso e o acolhimento. Ainda assim, a adoção dessas tecnologias requer atenção aos aspectos organizacionais e formativos. Dados indicam um uso crescente de telessaúde e plataformas como a Rede Universitária de Telemedicina (RUTE) e o Sistema Universidade Aberta do Sistema Único de Saúde (UNA-SUS), mas também revelam que menos da metade das UBS possuem infraestrutura física adequada para uso efetivo dessas ferramentas. Isso reforça a necessidade de investimento em equipamentos, conectividade e formação contínua dos profissionais para implementar com sucesso as tecnologias digitais em estratégias de continuidade do cuidado^(62)^. Assim, esta abordagem poderá contribuir para o gerenciamento longitudinal do cuidado, promover a continuidade informacional e fortalecer a coordenação entre os pontos da rede.

Apresentam-se, também, desafios relacionados ao vínculo paciente-profissional^(42,54)^, responsabilização das equipes da APS^(43)^ e envolvimento do paciente com seu projeto terapêutico^(27)^. Estes elementos estão imbricados, posto que o vínculo viabiliza a corresponsabilidade e a continuidade do cuidado, constituindo uma estratégia fundamental para fortalecer a relação terapêutica^(63)^.

A experiência de organização das RAS da região leste do Distrito Federal, a partir da Planificação da Atenção à Saúde, demonstrou que práticas colaborativas, escuta qualificada e valorização do saber do usuário são condições necessárias para a corresponsabilização no cuidado. Isso exige desenvolvimento de competências além do domínio técnico, incluindo a habilidade de dialogar, negociar metas e envolver o paciente no processo de tomada de decisão^(64)^.

A insuficiência e rotatividade de profissionais médicos na APS apresenta efeitos negativos no estabelecimento de vínculo com os usuários e continuidade do cuidado^(48,52,63)^. Por outro lado, pacientes hipertensos que mantêm acompanhamento com o mesmo médico por um período de dois anos apresentam uma qualidade de vida significativamente superior em comparação àqueles que recebem cuidados de múltiplos profissionais. O vínculo entre paciente e o profissional não apenas favorece uma maior adesão ao tratamento, mas também aumenta a disposição em seguir as orientações fornecidas^(11)^.

Os achados ainda destacam que visitas de pacientes a profissionais não habituais podem representar perda da continuidade e da coordenação do cuidado, além de frequentemente aumentar o quantitativo de encaminhamentos para a atenção especializada, uma vez que estes profissionais não conhecem o histórico clínico e não desenvolveram uma relação de cuidado continuado^(10)^.

Apesar disso, a elevada rotatividade dos profissionais, observada com frequência, limita a qualidade e disponibilidade do atendimento, constituindo barreira ao acesso e fragilizando o vínculo profissional-usuário^(65,66–67)^. Destaca-se, então, a necessidade de políticas que priorizem a estabilidade do profissional na APS e sua fixação no território.

A ausência de uma carreira estruturada, com estabilidade e progressão funcional consolidadas, aprofunda a rotatividade profissional, fragiliza o vínculo com os usuários e desarticula a lógica das RAS, além de reforçar relações precárias e terceirizadas de trabalho na saúde, com forte impacto na descontinuidade do cuidado^(68)^.

Frente a isso, reforça-se a necessidade de fortalecimento da APS, com investimentos estruturantes que envolvam o financiamento adequado, estímulo às ações intersetoriais, valorização do trabalho em saúde, implementação de políticas de manutenção de profissionais qualificados – como a instituição de uma carreira pública no SUS, medida defendida por movimentos sociais e pesquisadores do campo da Reforma Sanitária como essencial para valorização do trabalho com vistas à consolidação de um SUS universal e de qualidade. Assim, uma APS forte é capaz de garantir a continuidade do cuidado aos seus usuários^(68)^.

Diante do exposto, observa-se que a efetivação da continuidade do cuidado na APS ao usuário com HAS está diretamente relacionada às questões macropolíticas que direcionam desde a organização, funcionamento e gestão dos sistemas de saúde^(69)^. Na realidade brasileira, a persistência do modelo biomédico hegemônico - centrado em procedimentos, doença e condições agudas- desconsidera a epidemia das condições crônicas em todo o mundo, e a APS reproduz a prática clínica de urgências e emergências^(69,70)^. Este cenário é agravado pelo subfinanciamento crônico e o desfinanciamento do SUS imposto pela Emenda Constitucional n° 95/2016^(70)^, que compromete a alocação eficiente de recursos e a sustentabilidade das ações^(67)^. Além disso, a formação dos profissionais de saúde, frequentemente pautada por abordagens fragmentadas do conhecimento, mecanicista e baseada no paradigma positivista^(71)^, dificulta a adoção e a consolidação depráticas interdisciplinares, centrada na pessoa, contínuas e coordenadas no sistema de saúde. Ademais, as desigualdades regionais e a ausência de políticas efetivas de fixação de profissionais nas regiões mais vulneráveis amplificam as iniquidades no acesso a serviços de qualidade.

A qualificação permanente das equipes da APS para desenvolvimento de um cuidado continuado e centrado na pessoa, pautada em diretrizes alinhadas aos princípios do SUS, deve promover o uso de protocolos clínicos atualizados, mas, sobretudo, do desenvolvimento de vínculo e responsabilização. Tais estratégias são indispensáveis para a superação da fragmentação do cuidado e mudança assistencial, contribuindo para a consolidação de práticas integrais e coordenadas e, por conseguinte, promovendo melhores condições de saúde à pessoa com HAS.

Como limites do estudo, destaca-se a heterogeneidade metodológica dos estudos incluídos, que impôs desafios à análise dos dados. Por outro lado, a escassez de estudos com experiências positivas da efetivação da continuidade limitou a identificação de facilitadores para o desenvolvimento da continuidade do cuidado.

## CONCLUSÃO

Este estudo mapeou a produção científica relacionada ao cuidado às pessoas com diagnóstico de HAS, identificando efeitos, limites e estratégias para efetivação da continuidade do cuidado, no contexto da APS.

Revelou-se a importância da continuidade do cuidado na APS para pessoas com HAS, destacando efeitos positivos no controle da pressão arterial, adesão ao tratamento, redução da morbidade e mortalidade, diminuição do uso de serviços hospitalares e dos custos com saúde. Pacientes acompanhados pelo mesmo profissional têm maior probabilidade de alcançar controle da pressão arterial e aderir ao tratamento, reduzindo o risco de doenças cardiovasculares e a frequência de hospitalizações. Além disso, a continuidade do cuidado melhora a qualidade de vida dos pacientes hipertensos, pois o vínculo com o mesmo profissional de saúde facilita a adesão ao tratamento e a aceitação das orientações.

Os estudos mapeados sinalizam a existência de limites importantes para garantir a continuidade do cuidado na APS. Entre os fatores limitantes estão a falta ou descontinuidade na oferta de medicamentos, equipamentos para rastreamento da HAS, como esfigmomanômetro, a fragmentação do cuidado, a comunicação ineficaz entre profissionais, além da ausência de estratégias para a organização do cuidado para atender às necessidades clínicas dos pacientes. A rotatividade dos profissionais de saúde e a falta de envolvimento dos pacientes no planejamento terapêutico dificultam a formação de vínculos e a responsabilização das equipes de saúde.

A compreensão desses limites e das estratégias identificadas possibilita o reconhecimento das fragilidades e a orientação de ajustes necessários para a melhoria da continuidade do cuidado à pessoa HAS na APS. Além disso, pode subsidiar propostas de intervenção nos territórios, visando à implementação de estratégias para o enfrentamento dos desafios identificados, bem como estimular novas investigações — especialmente estudos longitudinais — que analisem os efeitos das estratégias adotadas na continuidade do cuidado e explicitem experiências exitosas relacionadas à prática da continuidade do cuidado na APS.

## Data Availability

Todo o conjunto de dados que dá suporte aos resultados deste estudo foi disponibilizado e publicado no próprio artigo.

# Translation: Continuidad de la atención a las personas con hipertensión en la atención primaria de salud: una revisión de alcance

**DOI:** 10.1590/1980-220X-REEUSP-2025-0053es

**Authors:** Amanda Maria Villas Bôas Ribeiro, Patty Fidelis de Almeida, Ana Luiza Queiroz Vilasbôas

**Affiliations:** 1 Universidade Federal da Bahia, Instituto de Saúde Coletiva, Salvador, BA, Brasil.; 2 Universidade Federal Fluminense, Instituto de Saúde Coletiva, Niteroi, RJ, Brasil.

**Keywords:** Hipertensión, Continuidad de la Atención al Paciente, Atención Primaria de Salud

## Abstract

**Objetivo:**

Mapear las evidencias sobre la continuidad de la atención a las personas con hipertensión arterial sistémica en la Atención Primaria de Salud (APS), señalando los límites y las estrategias para su implementación.

**Método:**

Revisión de alcance, sin delimitación temporal, a partir de las bases de datos Web of Science, PubMed, Literatura Latinoamericana y del Caribe en Ciencias de la Salud y Scientific Electronic Library Online. Se incluyeron tesis y disertaciones de la Biblioteca Digital Brasileña y del Catálogo de la Coordinación de Perfeccionamiento del Personal de Nivel Superior. La selección de los trabajos fue realizada por dos revisoras independientes y a ciegas, y las divergencias fueron resueltas por una tercera, lo que dio como resultado un corpus de 32 estudios. La extracción de los datos se realizó basándose en la adaptación del instrumento JBI, que abarca información sobre los estudios, la metodología y las recomendaciones para la continuidad de la atención. Los datos se organizaron en una hoja de cálculo Excel y se analizaron temáticamente, interpretándose a la luz de las dimensiones de la continuidad de la atención.

**Resultados:**

La continuidad de la atención en la hipertensión en la APS mejora el control de la presión arterial, reduciendo la morbilidad, la mortalidad y los costos de salud. Los pacientes atendidos por el mismo profesional de salud presentan una mejor adherencia al tratamiento y calidad de vida. Se identificaron limitaciones como la rotación de profesionales, la fragilidad en la comunicación entre servicios y la ausencia de flujos de atención consolidados. Como estrategias, destacan el fortalecimiento del trabajo en equipo, la articulación interprofesional, el uso de historias clínicas electrónicas compartidas y la adopción de flujos formales de referencia y contrarreferencia.

**Conclusión:**

Implementar una atención basada en el equipo y promover el intercambio de actividades entre los profesionales son estrategias para lograr la continuidad de la atención.

## INTRODUCCIÓN

En las últimas tres décadas, el número de personas con hipertensión arterial sistémica (HAS) en el mundo pasó de 650 millones en 1990 a 1300 millones en 2019, con estimaciones de prevalencia global del 49% entre hombres y mujeres de 50 a 79 años^(1)^, lo que evidencia su magnitud. Ante esta xsproblemática, es fundamental garantizar la adherencia al tratamiento farmacológico y no farmacológico, mediante el acceso a los servicios de salud, una buena relación con los profesionales^(2)^ y la continuidad de la atención para prevenir los riesgos y agravamientos derivados de la HAS, especialmente la aparición de isquemia miocárdica, enfermedad renal crónica y accidente vascular cerebral.

La Atención Primaria de Salud (APS), de base territorial y orientación comunitaria, desempeña un papel crucial en el control de las enfermedades crónicas no transmisibles, como en el caso de la HAS, y debe garantizar el acceso oportuno y adecuado a los servicios de la Red de Atención de Salud (RAS), coordinando la atención, interconectando los recursos comunitarios y asistenciales, para garantizar la continuidad de la atención sanitaria^(3)^.

Sin embargo, las personas con HAS tienen dificultades para acceder a los servicios de salud^(4,5)^, incluso en la APS, a pesar de su importancia para el control de la enfermedad y la reducción de la mortalidad^(6–8)^. Los retos a los que se enfrentan incluyen desde el acceso restringido y fragmentado a la atención^(9)^, la falta de recursos materiales y la disponibilidad de medicamentos^(6)^, hasta la falta de comunicación entre los profesionales de los servicios de salud^(10)^.

Ante la contatación de estos obstáculos, se hace evidente la importancia social de ampliar el debate sobre la continuidad de la atención para crear estrategias adecuadas para su implementación en la RAS bajo la coordinación de la APS, ya que el seguimiento regular y continuo del paciente con HAS reduce las posibilidades de complicaciones graves y mejora la adherencia terapéutica, el autocuidado y la calidad de vida^(11)^, reduce la sobrecarga de la media y alta complejidad con hospitalizaciones, exámenes y consultas^(12–14,15)^.

La continuidad de la atención se confunde a menudo con la longitudinalidad, la coordinación o incluso la integralidad de la atención^(16)^. Sin embargo, estos conceptos, aunque relacionados, presentan diferencias. La continuidad se considera como la oferta de cuidados integrados, coordinados y secuenciales a lo largo del tiempo, a partir del establecimiento de un vínculo y una confianza, centrados en las necesidades de salud del paciente, con la gestión de la información clínica compartida por diferentes profesionales y servicios de salud, bajo la coordinación de la APS^(16,17)^. Cabe destacar que la continuidad está vinculada a la experiencia individual de cada usuario en el transcurso de su proyecto terapéutico relacionado con un problema de salud específico^(18)^, como la HAS.

El objetivo del artículo es mapear las evidencias sobre el desarrollo de la continuidad de la atención a las personas con diagnóstico de HAS por la APS, señalando los límites y las estrategias para su implementación. Se realizó una búsqueda preliminar con los Medical Subject Headings (MeSH) «continuidad de la atención» e «hipertensión» en el Open Science Framework, Joanna Briggs Institute Evidence Synthesis, sin encontrar estudios o protocolos de revisión de alcance sobre el tema, lo que justifica la relevancia del artículo.

## MÉTODO

### Diseño del Estudio

Se trata de una revisión de alcance realizada según el método recomendado por el Joanna Briggs Institute (JBI)^(19)^ y presentada según las recomendaciones de la checklist Preferred Reporting Items for Systematic reviews and Meta-Analyses extension for Scoping Reviews (PRISMA-ScR)^(20)^.

Este tipo de investigación consiste en una revisión exploratoria destinada a mapear la literatura relevante en el área de interés y sus conceptos clave, así como a identificar lagunas en la literatura científica que ayuden a definir cuestiones de investigación^(21)^. Las etapas de la revisión de alcance incluyen: formulación de la pregunta y el objetivo de la investigación; identificación de documentos relevantes que se ajusten al alcance y la amplitud de los propósitos de la revisión; selección de los estudios según los criterios predefinidos; mapeo de datos; resumen de los resultados, mediante un análisis temático cualitativo en relación con el objetivo y la pregunta; y presentación de los resultados^(19)^.

El protocolo de esta revisión, diseñado a partir de la pregunta de investigación, se registró en la plataforma para el registro de trabajos científicos Open Science Framework a través del enlace osf.io/tdr6y.

### Pregunta Orientadora

La pregunta de investigación se delineó basándose en la estrategia mnemónica Problema, Concepto y Contexto (PCC). Esta metodología ayuda a identificar los temas clave para orientar la recopilación de datos.

En cuanto a los participantes (P), se definieron como personas con diagnóstico de HAS. En cuanto al concepto (C), se incluyeron en esta revisión estudios que abordan la continuidad de la atención a las personas con hipertensión arterial sistémica (HAS). El concepto de continuidad de la atención implica la relación establecida entre el profesional de la salud y el paciente que se prolonga más allá de los episodios de enfermedad, considerándose un «reflejo de un sentido de afiliación, a menudo expresado en términos de un contrato implícito de lealtad por parte del paciente y responsabilidad clínica por parte del médico», teniendo como prerrequisito el acceso a los servicios de salud y la coordinación de la atención como agente facilitador^(17)^. Y, a su vez, en lo que respecta al Contexto (C), se seleccionaron estudios realizados en la Atención Primaria de Salud, con alcance nacional, regional, municipal, distrital o local.

De este modo, conciliando los temas clave del PCC y el objetivo del estudio, la pregunta de investigación es: ¿Cómo se desarrolla la continuidad de la atención a las personas con diagnóstico de HAS por parte de la APS?

### Estrategias de Búsqueda

Esta revisión se realizó en las siguientes bases de datos: Web of Science Core Collection, PubMed a través de la Biblioteca Nacional de Medicina/Centro Nacional de Información Biotecnológica (NLM/NCBI), Literatura Latinoamericana y del Caribe en Ciencias de la Salud (LILACS) a través de la Biblioteca Virtual en Salud y la Scientific Electronic Library Online (SciELO). La búsqueda de literatura gris se realizó a través de la Biblioteca Digital Brasileña de Tesis y Disertaciones y el Catálogo CAPES.

Se utilizaron términos libres asociados a los descriptores de Medical Subject Headings (MeSH) y Descriptores en Ciencias de la Salud (DeCS): Primary Health Care, Hypertension y Continuity of patient care. Las estrategias de búsqueda siguieron las definiciones de la base de datos correspondiente y se desarrollaron con el apoyo de un bibliotecario especializado en revisiones. La recopilación de datos se realizó en enero de 2024.

### Criterios de Elegibilidad

De acuerdo con las recomendaciones^(22)^, se definieron criterios de inclusión y exclusión para la selección de los artículos. Así, las fuentes de evidencia de esta revisión fueron textos completos de estudios primarios, tesis y disertaciones, todos publicados en bases de datos del área de la salud. Se incluyeron estudios que abordaban la continuidad de la atención a personas con HAS, con documentos disponibles en su totalidad, publicados en español, portugués o inglés, sin límites de tiempo definidos, con el fin de recuperar el mayor número posible de publicaciones relacionadas con el tema de investigación. No se incluyeron artículos de revisión, protocolos de estudios de revisión, artículos de opinión, actas de congresos, comentarios, editoriales ni capítulos de libros.

### Extracción de Datos

Los artículos encontrados en la búsqueda se agruparon e importaron a la herramienta Rayyan Intelligent Systematic Review para excluir duplicados y seleccionar los trabajos. A continuación, dos revisoras realizaron de forma independiente y ciega la selección de las fuentes de evidencia a partir de la lectura de los títulos y resúmenes. Las discrepancias encontradas fueron resueltas por una tercera revisora y se justificaron los motivos de exclusión de los manuscritos. Posteriormente, los estudios que cumplían los criterios fueron evaluados en su totalidad para una apreciación completa del material retenido y la composición de la muestra final para la extracción de datos.

La extracción de datos se organizó a partir de la adaptación del instrumento desarrollado por el JBI^(20)^ para cumplir con los objetivos de este estudio, que contiene información sobre la identificación de los autores, el título completo, el año de publicación, los objetivos, la metodología (tipo de estudio, métodos adoptados, participantes y/o fuentes de datos, escenario, alcance), síntesis de los resultados, dificultades y facilidades para el desarrollo de la continuidad de la atención a personas con HAS en el ámbito de la APS y recomendaciones del estudio para lograr la continuidad de la atención. Todas las etapas de la extracción de datos de los estudios seleccionados se realizaron utilizando una hoja de cálculo Excel.

### Análisis y Presentación de los Datos

Para describir los resultados de la búsqueda y selección de los estudios, se utilizó el diagrama de flujo de identificación, escaneo y proceso de inclusión adaptado de los *Preferred Reporting Items for Systematic Review and Meta-analyses*(PRISMA-ScR)^(19)^.

Tras la selección de los estudios, los datos extraídos se organizaron en un instrumento de recopilación adaptado^(20)^, que contenía información como autores, año, país, objetivos, diseño metodológico, principales hallazgos y aspectos relacionados con la continuidad de la atención. El análisis de los datos se llevó a cabo mediante el análisis temático del contenido^(23)^, aplicado de forma sistemática a los datos cualitativos y cuantitativos.

En los estudios cuantitativos, los hallazgos numéricos se interpretaron a la luz de sus significados e implicaciones descritos en los propios artículos, y se categorizaron de acuerdo con las dimensiones temáticas emergentes, tales como barreras, efectos y estrategias asociadas a la continuidad de la atención. Así, los datos cuantitativos no se analizaron estadísticamente, sino que se integraron en el análisis cualitativo en función de su contenido interpretativo, según su relevancia para el objeto de este estudio.

El proceso de análisis siguió las siguientes etapas: (1) lectura fluida de los textos completos; (2) codificación de los datos extraídos en el instrumento de recolección; (3) categorización de los contenidos por similitud temática; (4) identificación de temas centrales relacionados con el objeto de la revisión, especialmente aquellos que trataban de los límites, efectos y estrategias para la efectividad de la continuidad de la atención.

Los resultados del análisis se organizaron y presentaron en cuadros explicativos, con síntesis narrativas, y se interpretaron a la luz de las dimensiones de la continuidad de la atención^(17)^. La continuidad de la atención puede entenderse a partir de tres dimensiones principales: administrativa, informativa y relacional. La continuidad gerencial se refiere a la gestión de la atención a lo largo del tiempo y de los diferentes puntos de la red de atención de salud, lo que exige la articulación entre los servicios y la gestión clínica adecuada a las necesidades del usuario^(17,18)^. La continuidad informativa permite el intercambio efectivo de información relevante sobre el historial, el contexto y la atención de salud del usuario entre los profesionales y los servicios^(16,17)^. Por último, la continuidad relacional implica el establecimiento de vínculos terapéuticos duraderos entre usuarios y profesionales de la salud, lo que sustenta la confianza, la acogida y la percepción de una atención continua a lo largo del tiempo^(17,18)^.

### Aspectos Éticos

Los datos utilizados son de acceso público, por lo que no fue necesario someterlos al Comité de Ética en Investigación.

## RESULTADOS

Inicialmente, se identificaron 230 estudios, de los cuales 214 eran artículos en bases de datos y 16 eran tesis y disertaciones. De estos estudios, 75 eran duplicados y, por lo tanto, fueron eliminados. Tras la lectura de los títulos y resúmenes realizada por dos investigadoras de forma doble ciego, se excluyeron 89 estudios por no tratar el tema o no cumplir los criterios establecidos. Se procedió a la lectura íntegra de 66 estudios, de los cuales se excluyeron 34, dos por no estar disponibles en su totalidad, siete por el tipo de publicación (actas, protocolos de estudios y directrices terapéuticas), 22 por no responder a la pregunta de investigación, dos por no tratar de la población en cuestión y uno por no estar relacionado con el concepto de continuidad de la atención.

Así, la muestra final estuvo compuesta por 32 estudios, de los cuales 20 eran artículos científicos y 12 tesis y disertaciones. Con el fin de sistematizar el proceso de selección de los estudios, se utilizó el PRISMA-SrC (Figura 1).

**Figura 1:** Fluxograma do processo de seleção dos estudos sobre continuidade do cuidado à pessoa com HAS no contexto da Atenção Pri-mária à Saúde.

A continuación, se presentó la caracterización de los estudios analizados y la síntesis de los hallazgos a partir de dos categorías generales identificadas a partir del análisis de contenido: efectos de la continuidad en la atención sanitaria de las personas con HAS y retos en el desarrollo de la continuidad de la atención al paciente con HAS en la APS.

### Caracterización de los Estudios

La dimensión temporal de los estudios correspondió al período comprendido entre 2005 y 2023, según la Cuadro 1. Se observa un incremento gradual de las publicaciones a partir de 2019, con alrededor del 52,94% de los estudios publicados entre los años 2019 y 2023. Predominaron los estudios cuantitativos (18 trabajos), especialmente los transversales (10 trabajos).

**Cuadro 1:** Caracterización de los estudios seleccionados según objetivos, metodología, idioma y año de publicación – Niterói, RJ, Brasil, 2025

Autores	País	Objetivo	Metodología
Inkster et al.^(24)^	Escocia	Investigar aspectos del tratamiento de la hipertensión en relación con variables sociodemográficas, tratamiento farmacológico antihipertensivo y factores organizativos en atención primaria.	Estudio observacional cuantitativo de 560 pacientes seleccionados aleatoriamente de entre 40 y 79 años que recibían tratamiento para la hipertensión. Se recogieron datos sobre el proceso asistencial.
Cunha^(25)^	Brasil	Investigar el cumplimiento del atributo de vinculación longitudinal en la experimentación de diferentes modelos organizacionales de atención a la APS en el contexto del SUS.	Estudio cualitativo realizado por medio de entrevistas semiestructuradas con profesionales, revisión de una muestra de historias clínicas y aplicación de encuesta a una muestra de usuarios.
Malfatti y Assunção^(8)^	Brasil	Evaluar el registro medio anual de diabetes e hipertensión y el correspondiente nivel de seguimiento de los pacientes por municipios.	Estudio transversal, utilizando los registros de diabetes e hipertensión y el seguimiento de los profesionales de salud de los equipos de APS de los municipios de una región sanitaria de Rio Grande do Sul.
Machado^(26)^	Brasil	Evaluar el grado de integración de la red de servicios de salud en la atención a personas con hipertensión.	Estudio evaluativo realizado con gestores y técnicos de atención primaria, vigilancia, cardiología, urgencias y emergencias, atención psicosocial, red hospitalaria y regulación.
Martins^(27)^	Brasil	Análisis del acceso y continuidad asistencial de pacientes con hipertensión y diabetes mellitus.	Estudio mixto con análisis documental, encuesta telefónica y entrevista semiestructurada. Se realizó un análisis comparativo según el tipo de unidad de salud: Unidades del Programa Médico de Familia, Unidades Básicas y Policlínicas.
Sousa et al.^(9)^	Brasil	Analizar las condiciones de acceso integral a la red asistencial, considerando desde la atención en la Unidad de Salud de la Familia hasta la atención especializada.	Estudio de métodos mixtos utilizando grupos focales con profesionales de APS y recogida de datos del Sistema Central de Regulación Sanitaria y del Sistema de Reserva de Cita Previa.
Katz et al.^(10)^	Canadá	Analizar las formas de acceso a la asistencia sanitaria para adultos con enfermedades crónicas.	Cohorte de 347.606 pacientes con al menos cuatro consultas externas con un médico de atención primaria o especialista durante el periodo de estudio.
Hanafi et al.^(28)^	Malasia	Investigar la relación entre la continuidad personal y el control de la presión arterial entre pacientes con hipertensión en un centro académico de atención primaria.	Estudio de cohortes longitudinal retrospectivo realizado mediante la revisión de 1.060 historias clínicas de pacientes adultos con hipertensión.
Li et al.^(29)^	China	Comparar la calidad percibida de la atención primaria en términos de accesibilidad, continuidad, coordinación y exhaustividad entre pacientes hipertensos.	Estudio transversal, utilizando la versión china adaptada y validada de la *Herramienta de Evaluación de la Atención Primaria* (*Primary Care Assessment Tool*-PCAT).
Li et al.^(30)^	China	Comparar la continuidad asistencial entre pacientes hipertensos entre el Modelo de Gestión Directa de los centros de salud comunitarios y el Modelo de Colaboración Suelta.	Estudio transversal realizado en 4 centros de salud de China aleatorizados a 386 pacientes en Nanjing y 396 en Wuhan.
Baricati^(31)^	Brasil	Comprensión de la longitudinalidad de la atención a partir de las experiencias de los usuarios de APS.	Estudio cualitativo, descriptivo y exploratorio realizado en el área urbana de un municipio.
Li et al.^(32)^	China	Investigar los cambios en la calidad percibida de la atención primaria por los pacientes hipertensos de edad avanzada.	Estudio transversal realizado en dos fases con pacientes atendidos en Atención Primaria de Salud en Shanghái.
Wollum et al.^(33)^	Sudáfrica	Identificar las características básicas y las barreras para el tratamiento de la hipertensión, la hipercolesterolemia y la diabetes.	Estudio de métodos mixtos con encuestas en unidades de APS, discusiones de grupos focales y entrevistas a informantes clave.
Santos^(34)^	Brasil	Evaluar la relación entre los servicios de atención primaria y los de atención especializada en la atención a los pacientes hipertensos.	Estudio cuanti-cualitativo realizado con médicos de atención primaria.
Khanam et al.^(35)^	Australia	Verificar la relación entre el control de la presión arterial y la continuidad asistencial.	Estudio cuantitativo, datos recogidos de 329 consultas generales ubicadas en toda Australia.
Lall et al.^(36)^	India	Comprender cómo se organiza la atención primaria de la diabetes y la hipertensión en los centros de salud de la India.	Estudio de métodos mixtos con entrevistas en profundidad a 24 pacientes con diabetes e hipertensión y 6 médicos en tres unidades sanitarias y tres unidades privadas.
Silva^(37)^	Brasil	Evaluar el efecto de la gestión de casos sobre la presión arterial y las complicaciones en adultos con hipertensión.	Ensayo clínico aleatorizado realizado en una unidad de salud de atención primaria.
Chukwuma et al.^(38)^	Tayikistán	Comprender las brechas en la continuidad de la atención en APS, los desafíos y proponer soluciones.	Estudio de métodos mixtos. Grupos focales con usuarios de servicios, proveedores de atención sanitaria y administradores.
Leniz e Gulliford^(39)^	Chile	Explorar factores relacionados con la continuidad de la atención y su asociación con la atención de diabetes e hipertensión y el control de la enfermedad.	Estudio transversal basado en datos de la Encuesta Nacional de Salud realizada en Chile con usuarios de APS.
Neves^(40)^	Brasil	Analizar cómo se configuran las relaciones entre los profesionales de la Estrategia de Salud de la Familia en la atención a usuarios con hipertensión y diabetes.	Estudio descriptivo con enfoque cualitativo y cuantitativo.
Barrera et al.^(41)^	Colombia	Identificar las condiciones asociadas al logro de hipertensión controlada.	Estudio observacional longitudinal con individuos diagnosticados con hipertensión atendidos en dos centros de APS.
Fraser-Hurt et al.^(6)^	Samoa	Evaluar los problemas en la continuidad de la atención e identificar posibles soluciones, utilizando la hipertensión como condición trazadora.	Estudio de métodos mixtos, utilizando datos de encuestas de hogares de adultos integrados con datos de registros médicos de pacientes con hipertensión y resultados de discusiones de grupos focales con pacientes y profesionales de la salud.
Xu et al.^(42)^	Hong Kong	Investigar la eficacia de la continuidad asistencial basada en equipos para prevenir la enfermedad cardiovascular y la mortalidad en pacientes con hipertensión.	Cohorte retrospectiva de pacientes que acudieron a atención primaria en Hong Kong de 2008 a 2018.
Bellas et al., 2023^(43)^	Brasil	Evaluar el desempeño resistente en términos de continuidad de la atención y atención longitudinal para la hipertensión arterial sistémica y la diabetes mellitus por las unidades de atención primaria durante la pandemia de Covid-19.	Se trata de un estudio de métodos mixtos con enfoque cuantitativo y cualitativo. Se utilizaron datos secundarios del Sistema de Información y entrevistas en profundidad con gestores y profesionales de gestión técnica de la coordinadora del área del programa del municipio de Río de Janeiro-Brasil.
Almalki et al.^(44)^	ArabiaSaudita	Explorar la continuidad asistencial y sus predictores en entornos de Atención Primaria de Salud para pacientes con enfermedades crónicas en Arabia Saudí.	Estudio transversal, realizado con 193 usuarios de APS con enfermedades crónicas que tuvieron al menos cuatro visitas previas a unidades de atención primaria en el último año.
Trivedi et al.^(45)^	Australia	Explorar los factores que pueden influir en el control de la presión arterial en pacientes con fibrilación auricular e hipertensión.	Estudio retrospectivo, transversal. Se utilizó una base de datos que incluía historias clínicas electrónicas de consultas generales.
Dehghani Tafti et al.^(46)^	Irán	Determinar la continuidad de la atención a pacientes con enfermedades crónicas como hipertensión y diabetes durante la pandemia de COVID-19.	Estudio transversal retrospectivo. La población del estudio estuvo formada por todos los pacientes que padecían diabetes tipo 2 e hipertensión y que tenían una historia clínica activa en uno de los centros de salud durante al menos dos años antes de la pandemia COVID-19.
Damião^(47)^	Brasil	Analizar el impacto de la pandemia en la continuidad asistencial de pacientes con HAS y Diabetes Mellitus en el municipio de Pinhais-PR.	Estudio longitudinal, retrospectivo, de 540 pacientes con hipertensión o diabetes, monitoreados por Unidades de Salud de la Familia.
Cardoso^(48)^	Brasil	Analizar la priorización de la atención para el combate al COVID-19 en relación a la atención sistemática de hipertensos, diabéticos, gestantes y niños en la Atención Primaria del Distrito Federal.	Estudio transversal, analizando datos de pacientes hipertensos, diabéticos, gestantes y niños seguidos en APS de 2018 a 2020, en el Distrito Federal.
Cavalcante(49)	Brasil	Analizar el seguimiento de pacientes con hipertensión durante la pandemia por la atención primaria.	Estudio descriptivo, cualitativo. Se utilizó la técnica de grupo focal con profesionales y gestores de salud.
Morais^(50)^	Brasil	Conocer las barreras y los facilitadores para las consultas y la medición de la presión arterial.	Estudio cualitativo, descriptivo, realizado con profesionales de Unidades Básicas de Salud.
Lopes^(51)^	Brasil	Comprender las repercusiones de la atención ofrecida a las personas que padecieron enfermedades crónicas durante la pandemia de COVID-19, ofrecida por el equipo del Programa Hiperdia.	Estudio cualitativo, con enfoque participativo, realizado con pacientes con hipertensión y diabetes y en seguimiento a distancia durante la pandemia.

Fuente: Elaboración propia.

Abreviaturas: APS: Atención Primaria de Salud; HAS: Hipertensión Arterial Sistémica.

De los estudios analizados, la mayoría eran municipales (25 estudios), seguidos de nacionales (4 estudios) y locales (3 estudios). En cuanto al lugar de realización de las investigaciones, a pesar de que el mayor número de estudios se encontró en países de América del Sur (Brasil, Chile, Colombia), con 17 producciones, sólo 5 eran artículos científicos y 12 tesis y tesinas. El continente asiático destacó en la producción científica analizada, con 9 artículos (Arabia Saudí, China, Corea del Sur, India, Irán, Hong Kong, Tayikistán, Malasia), seguido de uno de América del Norte (Canadá), uno de Europa (Escocia), tres de Oceanía (Australia y Samoa) y uno del continente africano (Sudáfrica).

### Efectos de la Continuidad Asistencial en Personas con Hipertensión Arterial Sistémica

Los efectos de la continuidad asistencial para las personas con hipertensión, según las categorías analíticas que surgieron del análisis de contenido, se muestran en la Cuadro 2.

**Cuadro 2:** Resumen de los efectos de la continuidad asistencial para personas con hipertensión arterial sistémica – Niterói, RJ, Brasil, 2025.

Categoría analítica	Causa/efecto
Control de la tensión arterial	Los pacientes que reciben atención del mismo médico tienen más probabilidades de lograr el control de la presión arterial^(35,41)^.
	El número de consultas aumenta las posibilidades de control de la presión arterial^(41)^.
Cumplimiento del tratamiento	El seguimiento regular de los pacientes por los mismos profesionales aumenta las probabilidades de adherencia al tratamiento^(11)^.
Reducción del daño en órganos diana	La continuidad asistencial se asocia positivamente con una reducción del riesgo global de enfermedad cardiovascular, cardiopatía coronaria e ictus^(42)^.
Utilización de servicios y gasto sanitario	La continuidad asistencial se asocia a una reducción de los costes/gastos sanitarios globales^(12,13)^ una reducción de la frecuencia de hospitalización y del uso de servicios hospitalarios (salas de urgencias, por ejemplo)^(14)^ un menor número de días de hospitalización y una reducción de los costes hospitalarios y ambulatorios^(15)^. Las zonas urbanas y rurales presentan diferencias en las tasas de hospitalización y el uso de servicios^(14)^.
Calidad de vida	Los pacientes tratados por los mismos profesionales sanitarios experimentan mejores niveles de calidad de vida^(11)^.
Comunicación paciente-profesional	Los profesionales sanitarios con altos niveles de continuidad asistencial son capaces de comunicarse mejor con sus pacientes, conocer su historial sanitario y su situación actual^(29)^.

Fuente: elaboración propia.

### Límites en el Desarrollo de la Continuidad Asistencial Para Pacientes con HAS en APS

Los límites de la continuidad de la atención a pacientes con HAS según cada dimensión de continuidad^(17)^ se sistematizan en la Cuadro 3.

**Cuadro 3:** Factores que limitan la continuidad asistencial de las personas con hipertensión arterial sistémica – Niterói, RJ, Brasil, 2025.

Dimensión de continuidad	Factores que limitan la continuidad
Continuidad de la gestión	Falta de puesta en común de las estrategias asistenciales y del proyecto terapéutico del paciente entre los profesionales de los distintos niveles asistenciales, ya sea a través de derivaciones y contrarrederivaciones o de reuniones entre equipos para discutir los casos^(44,52,53)^.
	Falta de estrategias para organizar la oferta/demanda en función de las necesidades clínicas de los pacientes con HAS(9).
	Falta de categorización del riesgo y de control de la presión arterial^(9,38)^.
	Largos tiempos de espera para la atención e insuficiente tiempo de consulta con el profesional^(9,27,33)^.
	Falta de acuerdo entre los servicios sanitarios de la zona para garantizar el flujo asistencial de los pacientes con HAS^(34)^.
	Insuficiente oferta de pruebas y consultas especializadas para el diagnóstico y control de la HAS^(36)^.
	Falta de recursos materiales para la aplicación y continuidad del tratamiento y seguimiento de los pacientes con HAS, especialmente de fármacos antihipertensivos^(6,33,36,54,55)^.
	Desconocimiento por parte de los profesionales de la APS del flujo asistencial de la HAS en la red municipal^(27)^.
	La insuficiencia de material, como el esfigmomanómetro, limita la capacidad de detección y seguimiento de la HAS^(33,36,54)^.
Continuidad de la información	Comunicación ineficaz/inexistente entre los profesionales de la APS y los servicios especializados^(9,52,55)^.
	Escasa integración entre los profesionales de los distintos niveles asistenciales de la red^(9,10)^.
	Proceso asistencial centrado en el médico y en los procedimientos, dificultando la comunicación e interacción entre los miembros del equipo^(36,48,52)^.
	Falta de cumplimentación adecuada de los formularios de derivación de pacientes con HAS para atención especializada^(27)^.
	Falta de sistematización y almacenamiento adecuado de los datos clínicos de los pacientes^(9,36)^. Historias clínicas incompletas e insuficientes^(16)^.
Continuidad relacional	Rotación de los profesionales del equipo de APS, lo que pone en peligro la formación de vínculos^(31,52)^.
	Número insuficiente de profesionales médicos en APS ante la creciente demanda de pacientes con HAS^(9,27,52)^.
	Falta de implicación de los usuarios en la toma de decisiones sobre su proyecto terapéutico^(36)^.
	Falta de compromiso/responsabilidad por parte de los equipos de Salud de Familia con el proyecto terapéutico del paciente^(34)^.

Fuente: Elaboración propia.

Abreviaturas: APS: Atención Primaria de Salud; HAS: Hipertensión Arterial Sistémica.

### Estrategias Para Garantizar la Continuidad Asistencial de los Pacientes con HAS

Aunque limitadas en número dada la complejidad del tema, las estrategias identificadas en la literatura para asegurar la continuidad asistencial de los pacientes con HAS se agruparon en función de las dimensiones de la atención, tal y como se sistematiza en la Cuadro 4. Estas estrategias reflejan iniciativas de dirigidas a asegurar la continuidad asistencial de los pacientes con HAS. Estas estrategias reflejan iniciativas de dirigidas a organizar la atención, mejorar la comunicación entre los servicios y reforzar los vínculos entre usuarios y profesionales.

**Cuadro 4:** Estrategias para realizar la continuidad de los cuidados según sus dimensiones – Niterói, RJ, Brasil, 2025.

Dimensión de la continuidad	Estrategias
Continuidad informativa	Establecimiento de un sistema de referencia y contrarreferencia entre niveles asistenciales^(29)^.
	Uso de las TIC, incluidos los servicios a distancia^(43)^.
	Adopción de herramientas normalizadas para evaluar y supervisar la continuidad asistencial^(11)^.
Continuidad de la gestión	Adopción del MACC para organizar la atención al HAS(36).
	Estructuración de los flujos asistenciales y organizativos formales en la APS(9).
	Educación sanitaria de los usuarios, centrada en la comprensión de la hipertensión y la adherencia al tratamiento(38).
Continuidad relacional	Formación de profesionales sanitarios centrada en la atención continua y centrada en el paciente(44).
	Participación de la comunidad en la reformulación de las prácticas sanitarias(46).

Fuente: Elaboración propia.

Leyenda: MACC: Modelo de Atención Crónica; TIC: Tecnologías de la Información y la Comunicación.

## DISCUSIÓN

Los resultados de este estudio señalan la importancia de la continuidad de la atención^(56)^ para las personas diagnosticadas con HAS. La continuidad de la atención no solo mejora la experiencia del paciente, sino que también tiene el potencial de mitigar las disparidades regionales en las tasas de morbilidad y mortalidad. Esto es especialmente relevante en contextos urbanos y rurales, donde las tasas de hospitalización relacionadas con HAS pueden variar considerablemente^(14)^.

Se identificaron varias limitaciones en el proceso de continuidad de la atención, especialmente en la integración entre los diferentes niveles de atención y la gestión de recursos^(6,33,36,38,54,55)^. La ausencia de estrategias eficaces de comunicación^(9)^ y de acuerdos entre los servicios y los profesionales, el desconocimiento de los flujos asistenciales^(27,34)^ y la insuficiencia de recursos materiales y humanos^(33)^ comprometen la calidad de la atención a las personas con HAS.

En este sentido, se señala la necesidad de estrategias de comunicación para fortalecer el sistema de referencia y contrarreferencia y los flujos asistenciales, el fortalecimiento de la integración entre los servicios y los profesionales de la asistencia y la implementación de una planificación integrada^(9)^. A esto se suma la escasa integración entre los profesionales de la APS y los hospitales para la elaboración de estrategias de control de la HAS, lo que pone de manifiesto la falta de colaboración interprofesional^(30)^. El proceso de atención centrado en la figura del médico, además de dificultar la interacción y el enfoque del tratamiento de la HAS, a menudo provoca una gran sobrecarga de atención a los profesionales médicos, lo que dificulta la participación del paciente en la toma de decisiones sobre el tratamiento y la división de tareas entre los miembros del equipo(36,48,52).

Un proceso de atención ofrecido por un equipo multidisciplinario puede promover la mejora de la continuidad de la atención, mediante una mejor coordinación y articulación entre sus miembros durante la práctica clínica^(42)^, además de promover el intercambio y la división de tareas^(53)^.

Cabe destacar también la falta de sistematización y organización de la información sobre los pacientes en la APS, o incluso la incompletitud de la información clínica. En muchos casos, los profesionales orientan a los usuarios a registrar manualmente el uso de medicamentos, el control de la presión arterial y otra información importante para el seguimiento, delegando en el propio individuo la responsabilidad de la continuidad informativa^(36)^.

Una estrategia relevante que se puede adoptar es la elaboración de planes de atención compartidos entre los diferentes niveles de atención, con el uso de tecnologías de la información y la comunicación que faciliten la transición de la atención y el intercambio de información clínica. Herramientas como los expedientes médicos electrónicos permiten el acceso integrado al proyecto terapéutico por parte de los profesionales, lo que favorece la corresponsabilidad en la atención, una mejor división de tareas en el equipo y la articulación entre los servicios^(33,46)^. Para su aplicación en el día a día de los servicios, se recomienda institucionalizar reuniones periódicas de los equipos multidisciplinarios con momentos dedicados a la elaboración conjunta de los planes, la definición de los objetivos asistenciales y el seguimiento de los casos.

La experiencia canadiense con las enfermeras de enlace pone de manifiesto la importancia de contar con profesionales con funciones específicas orientadas a la transición de la atención, como la planificación del alta hospitalaria y la transferencia segura de información clínica entre servicios. Estas enfermeras desarrollan competencias como el juicio clínico, el liderazgo y la comunicación, actuando como facilitadoras de la continuidad de la atención, especialmente en los casos más complejos, y apoyando una práctica centrada en las necesidades y potencialidades de los pacientes. Por su parte, la experiencia de Portugal destaca el fortalecimiento de la articulación interprofesional y el enfoque en la continuidad de la atención mediante la implementación de planes individualizados, elaborados con la participación de los usuarios y basados en una comunicación eficiente entre los diferentes niveles de atención^(57)^.

Además, la frágil comunicación entre los profesionales de la salud y los pacientes tuvo implicaciones directas en la continuidad, la adherencia al tratamiento y el control de la HAS^(6)^. Se debe aspirar a mejorar la comunicación con la adopción de un lenguaje más sencillo, sin terminología técnica, para facilitar la comprensión por parte de los usuarios^(29)^.

La inexistencia de una categorización del riesgo y un control adecuado de la presión arterial^(38)^, unida a los largos tiempos de espera, revela barreras significativas para la continuidad y la resolución de la atención, lo que se traduce en un agravamiento de la enfermedad, restricción del acceso, dificultades en la evaluación de la adherencia al tratamiento y falta de acogida. Estos factores culminan en inseguridad, discontinuidad y gastos financieros excesivos para los usuarios y sus familias^(58)^.

Estos hallazgos reflejan amplias dificultades en la implementación del modelo de atención basado en la APS, debido a la valoración del modelo de atención programática, aún predominante en los servicios de salud, con enfoque en la enfermedad y el tratamiento de afecciones agudas o el agravamiento de enfermedades crónicas, en la demanda espontánea y en acciones curativas y rehabilitadoras. En este contexto, el trabajo de los profesionales se centra en la enfermedad, la discapacidad y la muerte^(59,60)^, lo que dificulta la continuidad y la coordinación de la atención, la atención integral y la promoción de la salud.

Las dificultades de acceso a los servicios de salud pueden limitar la continuidad de la atención a las personas con HAS, ya sea por las distancias geográficas que dificultan la realización de consultas y la medición de la PA^(9,38)^, ya sea por las barreras que se encuentran para la realización de exámenes y consultas de seguimiento^(9,27)^. En este contexto, es fundamental adoptar estrategias que optimicen el acceso y la organización de la agenda asistencial, principalmente con el uso de tecnologías digitales^(51)^. La incorporación de tecnologías digitales, por ejemplo, ha demostrado ser eficaz en el contexto de la pandemia y puede mantenerse como recurso complementario para el seguimiento de personas con HAS, especialmente en zonas remotas o con escasez de profesionales^(43)^. Además, el uso de aplicaciones de programación en línea, el envío de recordatorios automáticos de consultas para reducir el absentismo^(61)^ y la ampliación de la oferta de horarios alternativos de atención son medidas que pueden implementarse en la práctica de la APS, contribuyendo a reducir las barreras de acceso y a fortalecer la continuidad de la atención.

En este contexto, cabe destacar la posibilidad de utilizar el sistema de citas en línea de la Historia Clínica del Ciudadano (PEC e-SUS APS), integrado en la aplicación *Conecte SUS Cidadão*, que permite a los pacientes ver los horarios disponibles, las agendas de citas e incluso las cancelaciones sin necesidad de desplazarse al centro de salud. Esta herramienta tiene el potencial de reducir el absentismo, mejorar la gestión de la agenda asistencial y ampliar el acceso y la resolución^(61)^.

Un estudio^(62)^ demostró que las unidades con historiales electrónicos tienen más posibilidades de funcionar como primer punto de contacto y de atender demandas urgentes, lo que pone de manifiesto que la informatización favorece el acceso y la acogida. Aun así, la adopción de estas tecnologías requiere prestar atención a los aspectos organizativos y formativos. Los datos indican un uso creciente de la telesalud y de plataformas como la Red Universitaria de Telemedicina (RUTE) y el Sistema Universitario Abierto del Sistema Único de Salud (UNA-SUS), pero también revelan que menos de la mitad de las UBS cuentan con la infraestructura física adecuada para el uso efectivo de estas herramientas. Esto refuerza la necesidad de invertir en equipos, conectividad y formación continua de los profesionales para implementar con éxito las tecnologías digitales en estrategias de continuidad de la atención^(62)^. Así, este enfoque puede contribuir a la gestión longitudinal de la atención, promover la continuidad informativa y fortalecer la coordinación entre los puntos de la red.

También se presentan desafíos relacionados con el vínculo entre el paciente y el profesional^(42,55)^, la responsabilidad de los equipos de APS^(38)^ y la participación del paciente en su proyecto terapéutico^(33)^. Estos elementos están interrelacionados, ya que el vínculo hace posible la corresponsabilidad y la continuidad de la atención, lo que constituye una estrategia fundamental para fortalecer la relación terapéutica^(63)^.

La experiencia de organización de las RAS de la región este del Distrito Federal, a partir de la Planificación de la Atención de Salud, demostró que las prácticas colaborativas, la escucha cualificada y la valoración del conocimiento del usuario son condiciones necesarias para la corresponsabilidad en la atención. Esto exige el desarrollo de competencias más allá del dominio técnico, incluyendo la capacidad de dialogar, negociar objetivos e involucrar al paciente en el proceso de toma de decisiones^(64)^.

La insuficiencia y la rotación de los profesionales médicos en la APS tienen efectos negativos en el establecimiento de vínculos con los usuarios y en la continuidad de la atención^(31,52,63)^. Por otro lado, los pacientes hipertensos que mantienen un seguimiento con el mismo médico durante un periodo de dos años presentan una calidad de vida significativamente superior en comparación con aquellos que reciben atención de múltiples profesionales. El vínculo entre el paciente y el profesional no solo favorece una mayor adherencia al tratamiento, sino que también aumenta la disposición a seguir las orientaciones proporcionadas^(11)^.

Los hallazgos también destacan que las visitas de los pacientes a profesionales no habituales pueden representar una pérdida de continuidad y coordinación de la atención, además de aumentar con frecuencia el número de derivaciones a la atención especializada, ya que estos profesionales no conocen el historial clínico y no han desarrollado una relación de atención continua^(10)^.

A pesar de ello, la elevada rotación de profesionales, que se observa con frecuencia, limita la calidad y la disponibilidad de la atención, lo que constituye una barrera para el acceso y debilita el vínculo entre el profesional y el usuario^(65–67)^. Cabe destacar, por tanto, la necesidad de políticas que prioricen la estabilidad del profesional en la APS y su permanencia en el territorio.

La ausencia de una carrera estructurada, con estabilidad y progresión funcional consolidadas, profundiza la rotación profesional, debilita el vínculo con los usuarios y desarticula la lógica de las RAS, además de reforzar las relaciones precarias y subcontratadas de trabajo en la salud, con un fuerte impacto en la discontinuidad de la atención^(68)^.

Ante esto, se refuerza la necesidad de fortalecer la APS, con inversiones estructurales que impliquen una financiación adecuada, el estímulo de acciones intersectoriales, la valorización del trabajo en salud y la implementación de políticas de mantenimiento de profesionales calificados, como la institución de una carrera pública en el SUS, medida defendida por movimientos sociales e investigadores del campo de la Reforma Sanitaria como esencial para la valorización del trabajo con vistas a la consolidación de un SUS universal y de calidad. Así, una APS fuerte es capaz de garantizar la continuidad de la atención a sus usuarios^(68)^.

Ante lo expuesto, se observa que la efectividad de la continuidad de la atención en la APS al usuario con HAS está directamente relacionada con las cuestiones macropolíticas que dirigen desde la organización, el funcionamiento y la gestión de los sistemas de salud^(69)^. En la realidad brasileña, la persistencia del modelo biomédico hegemónico —centrado en procedimientos, enfermedades y afecciones agudas— ignora la epidemia de afecciones crónicas en todo el mundo, y la APS reproduce la práctica clínica de urgencias y emergencias^(69,70)^. Este escenario se ve agravado por el subfinanciamiento crónico y el desfinanciamiento del SUS impuesto por la Enmienda Constitucional n.º 95/2016^(70)^, que compromete la asignación eficiente de recursos y la sostenibilidad de las acciones^(67)^. Además, la formación de los profesionales de la salud, a menudo basada en enfoques fragmentados del conocimiento, mecanicistas y basados en el paradigma positivista^(71)^, dificulta la adopción y consolidación de prácticas interdisciplinarias, centradas en la persona, continuas y coordinadas en el sistema de salud. Por otra parte, las desigualdades regionales y la ausencia de políticas eficaces para fijar a los profesionales en las regiones más vulnerables amplifican las inequidades en el acceso a servicios de calidad.

La cualificación permanente de los equipos de APS para el desarrollo de una atención continua y centrada en la persona, basada en directrices alineadas con los principios del SUS, debe promover el uso de protocolos clínicos actualizados, pero, sobre todo, el desarrollo de vínculos y la responsabilización. Estas estrategias son indispensables para superar la fragmentación de la atención y el cambio asistencial, contribuyendo a la consolidación de prácticas integrales y coordinadas y, por consiguiente, promoviendo mejores condiciones de salud para las personas con HAS.

Como limitaciones del estudio, cabe destacar la heterogeneidad metodológica de los estudios incluidos, lo que planteó retos para el análisis de los datos. Por otro lado, la escasez de estudios con experiencias positivas en la implementación de la continuidad limitó la identificación de facilitadores para el desarrollo de la continuidad de la atención.

## CONCLUSIÓN

Este estudio mapeó la producción científica relacionada con la atención a personas con diagnóstico de HAS, identificando efectos, límites y estrategias para la efectividad de la continuidad de la atención, en el contexto de la APS.

Se reveló la importancia de la continuidad de la atención en la APS para personas con HAS, destacando efectos positivos en el control de la presión arterial, la adherencia al tratamiento, la reducción de la morbilidad y la mortalidad, la disminución del uso de servicios hospitalarios y los costos de salud. Los pacientes atendidos por el mismo profesional tienen más probabilidades de controlar la presión arterial y adherirse al tratamiento, lo que reduce el riesgo de enfermedades cardiovasculares y la frecuencia de hospitalizaciones. Además, la continuidad de la atención mejora la calidad de vida de los pacientes hipertensos, ya que el vínculo con el mismo profesional de salud facilita la adherencia al tratamiento y la aceptación de las orientaciones.

Los estudios analizados señalan la existencia de importantes limitaciones para garantizar la continuidad de la atención en la APS. Entre los factores limitantes se encuentran la falta o la discontinuidad en el suministro de medicamentos, los equipos para el control de la HAS, como el esfigmomanómetro, la fragmentación de la atención, la comunicación ineficaz entre los profesionales, además de la ausencia de estrategias para la organización de la atención que satisfaga las necesidades clínicas de los pacientes. La rotación de los profesionales de la salud y la falta de participación de los pacientes en la planificación terapéutica dificultan la formación de vínculos y la responsabi-lización de los equipos de salud.

La comprensión de estas limitaciones y de las estrategias identificadas permite reconocer las debilidades y orientar los ajustes necesarios para mejorar la continuidad de la atención a las personas con HAS en la APS. Además, puede respaldar propuestas de intervención en los territorios, con el objetivo de implementar estrategias para hacer frente a los retos identificados, así como estimular nuevas investigaciones —especialmente estudios longitudinales— que analicen los efectos de las estrategias adoptadas en la continuidad de la atención y expliquen experiencias exitosas relacionadas con la práctica de la continuidad de la atención en la APS.

## Data Availability

Todo el conjunto de datos que respalda los resultados de este estudio se puso a disposición y se publicó en el propio artículo.
